# A Tau Pathogenesis-Based Network Pharmacology Approach for Exploring the Protections of *Chuanxiong Rhizoma* in Alzheimer’s Disease

**DOI:** 10.3389/fphar.2022.877806

**Published:** 2022-04-21

**Authors:** Peng Zeng, Hong-Fei Su, Chao-Yuan Ye, Shuo-Wen Qiu, Anbing Shi, Jian-Zhi Wang, Xin-Wen Zhou, Qing Tian

**Affiliations:** ^1^ Department of Pathology and Pathophysiology, School of Basic Medicine, Tongji Medical College, Key Laboratory of Neurological Disease of National Education Ministry, Huazhong University of Science and Technology, Wuhan, China; ^2^ Department of Biochemistry and Molecular Biology, School of Basic Medicine, Tongji Medical College, Cell Architecture Research Institute, Huazhong University of Science and Technology, Wuhan, China

**Keywords:** *Chuanxiong Rhizoma*, Alzheimer’s disease, phytochemicals, tau pathogenesis, mechanism

## Abstract

Alzheimer’s disease (AD) is the most common cause of neurodegenerative dementia and one of the top medical concerns worldwide. Currently, the approved drugs to treat AD are effective only in treating the symptoms, but do not cure or prevent AD. Although the exact causes of AD are not understood, it is recognized that tau aggregation in neurons plays a key role. *Chuanxiong Rhizoma* (CR) has been widely reported as effective for brain diseases such as dementia. Thus, we explored the protections of CR in AD by a tau pathogenesis–based network pharmacology approach. According to ultra-HPLC with triple quadrupole mass spectrometry data and Lipinski’s rule of five, 18 bioactive phytochemicals of CR were screened out. They were shown corresponding to 127 tau pathogenesis–related targets, among which VEGFA, IL1B, CTNNB1, JUN, ESR1, STAT3, APP, BCL2L1, PTGS2, and PPARG were identified as the core ones. We further analyzed the specific actions of CR-active phytochemicals on tau pathogenesis from the aspects of tau aggregation and tau-mediated toxicities. It was shown that neocnidilide, ferulic acid, coniferyl ferulate, levistilide A, Z-ligustilide, butylidenephthalide, and caffeic acid can be effective in reversing tau hyperphosphorylation. Neocnidilide, senkyunolide A, butylphthalide, butylidenephthalide, Z-ligustilide, and L-tryptophan may be effective in promoting lysosome-associated degradation of tau, and levistilide A, neocnidilide, ferulic acid, L-tryptophan, senkyunolide A, Z-ligustilide, and butylidenephthalide may antagonize tau-mediated impairments of intracellular transport, axon and synaptic damages, and neuron death (especially apoptosis). The present study suggests that acting on tau aggregation and tau-mediated toxicities is part of the therapeutic mechanism of CR against AD.

## Introduction

*Conioselinum anthriscoides* “Chuanxiong” (rhizome), also known as *Chuanxiong Rhizoma* (CR), is one of the most important herbs promoting the movement of *qi* and blood. CR has been widely used to dispel the stasis of blood, release the stagnation of *qi,* drive out *feng*, and relieve pain (Cai et al., 2014; Chen et al., 2018). According to the literature, there were about 100 prescriptions out of 520 Chinese patent medicines containing CR to treat encephalopathy (Zheng et al., 2013). The dried rhizome of CR is the main component of several traditional Chinese medicine decoctions with preventive effects on dementia, such as *Chuanxiong Tianma San* (川芎天麻散), *Taohong Siwu Decoction* (桃红四物汤), and *Yufeng Decoction* (愈风汤). From 104 kinds of traditional Chinese medicine decoctions for the treatment of senile dementia, 147 Chinese herbs were identified, and the medicine pair most used was CR and *Acorus tatarinowii* (Zong et al., 2014; Yang et al., 2017). It was also shown that CR is a common monarch herb for the treatments of Alzheimer’s disease (AD) and vascular dementia (Han et al., 2014; Wen et al., 2018).

As the most common cause of neurodegenerative dementia and one of the top medical concerns worldwide, AD is characterized by extracellular amyloid-β (Aβ) plaques, intracellular tau aggregation into neurofibrillary tangles (NFTs), neuronal synaptic dysfunction, and neuronal loss (Hinz and Geschwind, 2017). Notably, deposition of tau was evident many years before clinical symptoms appeared (Quiroz et al., 2018), and the extent of tau lesions in the brain has been shown to be closely related to the severity of dementia in AD patients (Braak and Braak, 1991). Hyperphosphorylation is one of the most prevalent posttranslational modifications found in aggregated tau isolated from the brains of AD patients. Thus, phosphorylated tau (p-tau) in cerebrospinal fluid has been used as a biomarker for the diagnosis of AD (Dubois et al., 2014; Xia et al., 2021). Cerebrospinal fluid p-tau such as that phosphorylated at Thr181 (p-tau181) or phosphorylated at Thr231 (p-tau231) has been significantly associated with neocortical NFT pathology and hippocampal atrophy rates (Hampel et al., 2005; Buerger et al., 2006). None of the pharmacologic treatments (drugs) available today for AD slow down or stop the damage and destruction of neurons that cause AD’s symptoms. There are currently six drugs used clinically to improve AD symptoms, such as cholinesterase inhibitors, N-methyl-D-aspartic acid (NMDA) receptor antagonist, and Aduhelm (aducanumab). Noteworthy, Aduhelm is AD’s first targeted therapy drug based on the Aβ hypothesis, which has received considerable suspicion and controversy (Lalli et al., 2021; Rabinovici, 2021). Since there are currently no drugs that can cure or prevent AD, it is of great significance to find effective phytochemicals in CR against the important pathological changes, such as tau aggregation, in AD.

The various bioactivities of CR are attributed to its phytochemicals. Among the numerous phytochemicals, aromatic acids, phthalides, and alkaloids are the major bioactive phytochemicals of CR (Donkor et al., 2016). Phthalides are characteristic components of quality control in CR, among which Z-ligustilide is often used as a marker compound to evaluate the quality of CR. Aromatic acids stimulate blood circulation and eliminate blood stasis by preventing platelet aggregation and acting as antithrombotic agents (Li et al., 2012). Tetramethylpyrazine (TMP), a characteristic alkaloid isolated from CR, was reported to reduce Aβ and tau pathology in several dementia animals (Lu et al., 2017; Huang et al., 2021). Ferulic acid has free radical scavenging and anti-inflammatory activities (Sakai et al., 1997; Zhang et al., 2003). Senkyunolide has anti-cyclooxygenase activity, and Z-ligustilide has antiproliferative activity (Kobayashi et al., 1992; Momin and Nair, 2002). Z-ligustilide also protects against Aβ_25-35_-induced neurotoxicity by regulating the tumor necrosis factor α (TNFα)–activated nuclear factor κB (NF-κB) signaling pathway (Kuang et al., 2009). Butylidenephthalide provides neuroprotection by reducing the release of various proinflammatory molecules from activated microglia (Nam et al., 2013). But generally, the phytochemicals of CR are numerous, and the pathogenesis of AD is very complex, making it difficult to catch the key phytochemicals and main action pathways of CR in AD treatment.

Overexpression, abnormal modifications, and degradation obstruction make tau protein accumulate in neurons, resulting in a series of neurotoxic effects, such as organelle dysfunctions, axon damages, synaptic loss and dysfunction, intracellular transport disorders, and neuronal death. Therefore, we applied a novel network pharmacology strategy to investigate the phytochemicals in CR with potential therapeutic effects against the above tau pathogenesis. To increase the credibility of the phytochemical data source, the bioactive phytochemicals of CR were obtained from recent studies based on ultra-high–performance liquid chromatography (UHPLC) with triple quadrupole mass spectrometry (UHPLC-MS/MS) (Yin et al., 2019; Wang et al., 2020). According to the UHPLC-MS/MS data and Lipinski’s rule of five (RO5), in CR, 18 bioactive phytochemicals corresponding to 127 tau pathogenesis–related targets were screened out. Furthermore, it was found that CR targets tau hyperphosphorylation, promotes tau lysosome–associated degradation, and antagonizes tau-mediated impairments of intracellular transport, axon and synaptic damages, and neuron death. This tau pathogenesis network pharmacology study partially reveals the mechanism of CR against AD.

## Materials and Methods

### Determination of the Main Phytochemicals of CR and Evaluation of Pharmacological Parameters

The composition of CR is complex and diverse. A total of 20 bioactive phytochemicals of CR were obtained from a recent study using UHPLC-MS/MS (Wang et al., 2020). In addition, TMP, a main bioactive phytochemical of CR, was also included (Zhu et al., 2021; Lin et al., 2021). The chemical structures in canonical SMILES format were extracted from the PubChem database (https://pubchem.ncbi.nlm.nih.gov/) (Kim et al., 2016). The 2D chemical structures were drawn with ChemDraw Ultra 8.0 software. RO5, i.e., molecule weight (MW) < 500, number of hydrogen bond donors (Hdon) ≤ 5, number of hydrogen bond acceptors (Hacc) ≤ 10, lipid–water partition coefficient (LogP) ≤ 5, and number of rotatable bonds (Rbon) ≤ 10, has been extensively used to evaluate bioavailability based on the structures of compounds. Here, we employed the SwissADME web tool (www.swissadme.ch) (Daina et al., 2017; Zeng et al., 2021a) to evaluate the compounds according to RO5. The main phytochemicals of CR consistent with RO5 were filtered out for the follow-up studies. The Traditional Chinese Medicine Systems Pharmacology Database and Analysis Platform (TCMSP) database (https://tcmsp-e.com/) (Ru et al., 2014) was used to evaluate the blood–brain barrier (BBB) score of the bioactive phytochemicals of CR.

### Evaluation of Toxicological Parameters

The contents of bioactive phytochemicals (mg/g) of CR were from a recent report (Wang et al., 2020), in which 36 CR dried rhizome samples from six different production origins (Dongpo, Meishan City; Dujiangyan City; Pengshan, Meishan City; Pengzhou City; Qionglai City; and Shifang City) in Sichuan Province, China, were tested. Drug toxicology is one of the key fields of preclinical research. The toxicological parameters of the identified CR bioactive phytochemicals were determined *via* Protox II webserver (https://tox-new.charite.de/protox_II/) (Banerjee et al., 2018). The toxicological end points (hepatotoxicity, carcinogenicity, immunotoxicity, and mutagenicity) are reported in a binary format as active or inactive. Synthetic accessibility score (SAscore) was calculated by the ADMETlab 2.0 (Xiong et al., 2021); a SAscore >6 implies that it is difficult to synthesize and a SAscore <6 implies easy to synthesize.

### Collection of CR–Related Targets

As a web server for target prediction of bioactive small molecules, the SwissTargetPrediction database (http://www.swisstargetprediction.ch/) (Daina et al., 2019; Zeng et al., 2021b) was used to collect the potential targets of the CR’s main phytochemicals. Specifically, the canonical SMILES was input into the SwissTargetPrediction, and the target species was set as *Homo sapiens*. Subsequently, the target information was collected and organized by the Microsoft Excel software (version 2019).

### Analysis of Genes Correlated With Tau Pathogenesis

The AlzData database (http://www.alzdata.org/), a widely used AD database that collects current high-throughput omics data (Xu et al., 2018), was used to analyze the relationship between the CR targets and tau pathogenesis. The CR potential targets were used as input into AlzData database (“CFG rank” module) for the correlation analysis with tau pathogenesis. The Microsoft Excel software (version 2019, Microsoft, Redmond, WA, United States) was then used to collate the obtained results. The normalized expression levels of the CR targets related to tau pathogenesis in the control and AD groups in the Gene Expression Omnibus (GEO) data set were analyzed with the “Differential expression” module of AlzData. The AlzData database contains gene expression data of different cell types from the human brain single-cell RNA-seq (GSE67835) and was used to identify the target-related cell type in this study. The GraphPad Prism software (version 8.0, San Diego, CA, United States) was used for graphical visualization. We further investigated the functional classification of the targets correlated with tau pathogenesis by the Panther classification system (http://pantherdb.org/) (Mi et al., 2007). The Sankey diagrams were plotted using the OriginPro 2021 software (OriginLab Corporation, Northampton, MA, United States).

### Protein–Protein Interaction Network Construction and Screening of Core Targets

The PPI network maintains the proper function of all organisms. The PPI network of the target proteins was constructed by the STRING database (version 11.5, https://string-db.org/) (Szklarczyk et al., 2019) and visualized by the Cytoscape software (version 3.7.1) (Shannon et al., 2003). By default, only PPIs with an interaction score exceeding the threshold of 0.4 (medium confidence) were included. In the PPI network, degree refers to the number of other nodes directly connected to a node. The core targets are defined as the targets in the top 20 ranked by the degree values calculated using Network Analysis (a Cytoscape plugin). The larger a node’s degree is, the more important the node is in the PPI network.

### Disease Ontology, Gene Ontology, and the Kyoto Encyclopedia of Genes and Genomes Pathway Enrichment Analysis

DO annotates genes are based on human disease, and GO terms are divided into three categories, namely, biological process (BP), cellular component (CC), and molecular function (MF). In this research, the DO, GO, and KEGG pathway enrichment analyses were performed by DOSE Bioconductor (Yu et al., 2015) and ClusterProfiler R package (version 3.12.0) (Yu et al., 2012). The *p* values were adjusted using the Benjamini–Hochberg (BH) method. The statistical significance was denoted if *p* value <0.05. Th rich factor is the ratio of the gene numbers to all gene numbers annotated in this term. The top 20 GO terms and KEGG pathways sorted by the *p* value were visualized by an online tool (http://www.bioinformatics.com.cn/) (Zeng et al., 2021c).

### Cell Culture and Proteomic Analysis

In our laboratory, 293 human embryonic kidney cells stably expressing human full-length tau (HEK293/tau) or the vector (HEK293/vec) were established (Li et al., 2007). The cells were cultured in Dulbecco’s Modified Eagle’s Medium (DMEM, Thermo Fisher Scientific, Waltham, MA, United States) supplemented with 10% fetal bovine serum (FBS, Gibco BRL, Gaithersburg, MD) and 200 mg/ml G418 (Thermo Fisher Scientific, Waltham, MA, United States), and were grown in 37°C in a humidified atmosphere of 5% CO_2_.

As has been indicated previously (Fang et al., 2018; Shi et al., 2020), the integrated approach to quantify the proteomic changes in HEK293/tau cells included isobaric tags for relative and absolute quantification (iTRAQ), HPLC fractionation, and MS-based quantitative proteomics. The proteins were digested by trypsin, and the obtained peptides were labelled by iTRAQ 6-plex reagents and fractionated by high pH reversed-phase HPLC. For HPLC/tandem MS analysis, the peptides were then dissolved in 0.1% (vol/vol) formic acid, loaded onto a reversed phase pre-column (Acclaim PepMap 100; Thermo Fisher Scientific) and separated by a reversed-phase analytical column (Acclaim PepMap RSLC; Thermo Fisher Scientific). The resulting peptides were analyzed by a Q Exactive™ Plus hybrid quadrupole–Orbitrap mass spectrometer (Thermo Fisher Scientific). To identify and quantify proteins, the resulting MS/MS data were analyzed by MaxQuant with an integrated Andromeda search engine, and the tandem mass spectra were searched against the UniProt human protein database. The differentially expressed proteins were defined as 1.3-fold changes (>1.30 or <0.77) and *p* < 0.05. The gene set enrichment analysis (GSEA) was performed using ClusterProfiler R package (version 3.12.0). For each GSEA, the normalized enrichment score (NES) and *p* value were specified. Using Venny 2.1 (https://bioinfogp.cnb.csic.es/tools/venny/index.html), the molecules present in both the HEK293/tau/HEK293/vec differentially expressed protein data set and CR target data set were screened out.

### Molecular Docking Simulations

Molecular docking is a computational method to study the interaction between the receptor and its ligands. The 3D molecular structures of CR’s key phytochemicals were retrieved from the PubChem database, and the structure files of the target proteins were acquired from the RCSB Protein Data Bank (PDB database, http://www.rcsb.org/) (Westbrook et al., 2002). The molecular docking was performed using the LeDock program (http://www.lephar.com/software.htm) (Wang Z. et al., 2016) with high accuracy in pose prediction. The receptor files were processed by the LePro tool, and all parameters were set to default for conformation sampling by a combination of simulated annealing and evolutionary optimization. The docking scores were calculated by the default scoring function. The PDB codes are: human CTNNB1 (2GL7), JUN (5FV8), ESR1 (1ERE), STAT3 (6NJS), BCL2L1 (3SP7), PTGS2 (5F19), PPARG (1FM9), NOTCH1 (2F8Y), CCND1 (2W96), TLR4 (3UL7), CXCL8 (1ILQ), MAPK1 (1PME), MAPK14 (1BL6), MMP2 (1CK7), GSK3B (2O5K), RELA (3QXY), and ICAM1 (1IC1).

## Results

### Main Bioactive Phytochemicals of CR and Their Pharmacological Properties

**FIGURE 1:**
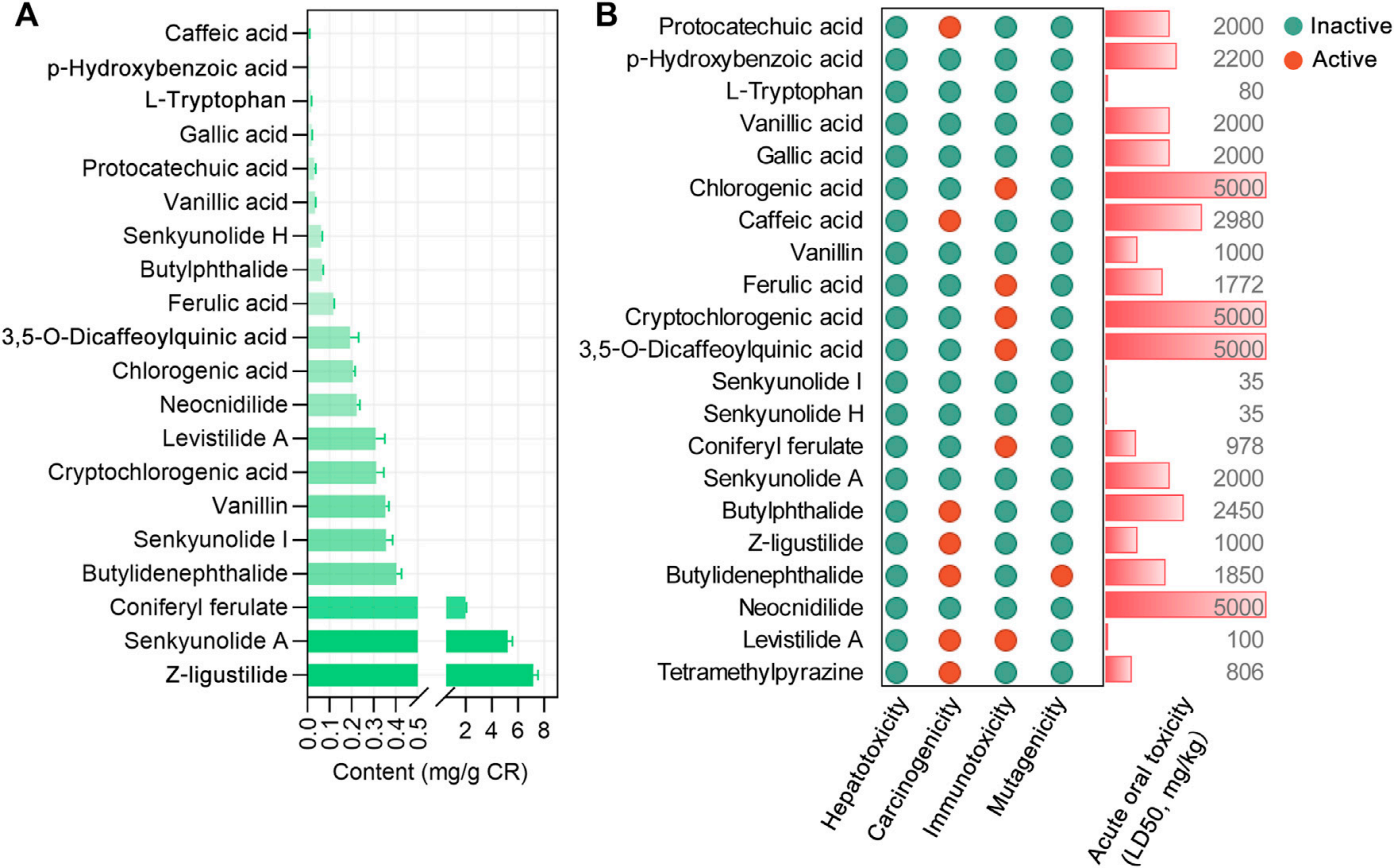
The content and toxicological parameters of bioactive phytochemicals in *Chuanxiong Rhizoma* (CR). **(A)** The contents of chemical constituents in CR dried rhizome samples from different origins in the Sichuan Province, China. The data were derived from in a recent report (Wang et al., 2020) and expressed as mean ± standard errors of the mean (SEM). **(B)** The toxicological parameters of 21 phytochemicals in CR, such as hepatotoxicity, carcinogenicity, immunotoxicity, mutagenicity, and acute oral toxicity (LD50, mg/kg). The red and green circles represent active or inactive toxicological end points, respectively.

We focused on 21 main bioactive phytochemicals of CR identified by UHPLC-MS/MS, namely, 12 aromatic acids, 8 phthalides, and TMP (Wang et al., 2020; Yin et al., 2019). According to the quantitative data (Wang et al., 2020), Z-ligustilide has the highest content (7.16 ± 0.36 mg/g CR), followed by senkyunolide A (5.2 ± 0.36 mg/g CR) and coniferyl ferulate (1.98 ± 0.08 mg/g CR) (Figure 1A). The content of TMP is very low (0. 60 to 11.75 μg/g CR) (Yin et al., 2018). A previous study (Li et al., 2014) also measured the content of nine phytochemicals in CR (Supplementary Figure S1). We utilized the SwissADME database (Daina et al., 2017; Zeng et al., 2021a) to calculate the physicochemical properties relevant to RO5. Three ingredients, chlorogenic acid, cryptochlorogenic acid, and 3,5-O-dicaffeoylquinic acid were shown to violate RO5 (Table 1). Using the SAscore to assess the ease of synthesis of drug-like molecules (Ertl and Schuffenhauer, 2009), it was shown that only levistilide A was difficult to synthesize (Table 1). The BBB score was evaluated and a score of ≥ −0.3 means that it is easy to cross the BBB. A large majority of the phytochemicals of CR can cross the BBB and enter the central nervous system, with senkyunolide A, neocnidilide, butylidenephthalide, Z-ligustilide, and TMP showing better BBB penetration. The prediction of the potential side effects of drugs is an important part of the drug development process. Hence, we used the Protox II webserver to analyze the febrile toxicological parameters of these 21 phytochemicals, such as hepatotoxicity, carcinogenicity, immunotoxicity, mutagenicity, and acute oral toxicity (LD50, mg/kg). All the bioactive phytochemicals of CR have no hepatotoxicity, and chlorogenic acid, cryptochlorogenic acid, 3,5-O-dicaffeoylquinic acid, and neocnidilide showed the highest LD50 values (Figure 1B). Since chlorogenic acid, cryptochlorogenic acid, and 3,5-O-dicaffeoylquinic acid violate RO5 (Table 1), the follow-up studies were conducted on the remaining 18 RO5-compliant CR phytochemicals.

**TABLE 1:** 21 Main phytochemicals in CR and their properties.

Compound	Formula	MW (g/mol)	Hdon	Hacc	Rbon	LogP	SAscore	BBB
Protocatechuic acid	C_7_H_6_O_4_	154.12	3	4	1	0.65	1.71	−0.17
p-Hydroxybenzoic acid	C_7_H_6_O_3_	138.12	2	3	1	1.05	1.38	0.21
L-Tryptophan	C_11_H_12_N_2_O_2_	204.23	3	3	3	0.17	2.30	−0.17
Vanillic acid	C_8_H_8_O_4_	168.15	2	4	3	1.08	1.54	0.09
Gallic acid	C_7_H_6_O_5_	170.12	4	5	1	0.21	2.10	−0.54
Chlorogenic acid	C_16_H_18_O_9_	354.31	6	9	5	−0.38	3.87	−1.71
Caffeic acid	C_9_H_8_O_4_	180.16	3	4	2	0.93	2.04	0.11
Vanillin	C_8_H_8_O_3_	152.15	1	3	2	1.2	1.82	0.41
Ferulic acid	C_10_H_10_O_4_	194.18	2	4	3	1.36	1.87	−0.03
Cryptochlorogenic acid	C_16_H_18_O_9_	354.31	6	9	5	−0.32	3.59	−1.73
3,5-O-Dicaffeoylquinic acid	C_25_H_24_O_12_	516.45	7	12	9	0.76	4.21	−2.04
Senkyunolide I	C_12_H_16_O_4_	224.25	2	4	2	1.17	4.24	0.5
Senkyunolide H	C_12_H_16_O_4_	224.25	2	4	2	1.18	4.24	—
Coniferyl ferulate	C_20_H_20_O_6_	356.37	2	6	8	3.25	2.34	−0.16
Senkyunolide A	C_12_H_16_O_2_	192.25	0	2	3	2.71	3.28	1.34
Butylphthalide	C_12_H_14_O_2_	190.24	0	2	3	2.81	2.56	—
Z-Ligustilide	C_12_H_14_O_2_	190.24	0	2	2	2.75	3.61	1.25
Butylidenephthalide	C_12_H_12_O_2_	188.22	0	2	2	2.94	2.45	1.27
Neocnidilide	C_12_H_18_O_2_	194.27	0	2	3	2.87	3.62	1.32
Levistilide A	C_24_H_28_O_4_	380.48	0	4	4	4.73	6.33	—
Tetramethylpyrazine	C_8_H_12_N_2_	136.19	0	2	0	0.03	1.97	1.05

MW, molecule weight; Hdon, number of hydrogen bond donors; Hacc, number of hydrogen bond acceptors; Rbon, number of rotatable bonds; LogP, lipid–water partition coefficient; SAscore, synthetic accessibility score; BBB, blood–brain barrier.

### Tau Pathogenesis–Associated Targets of CR's Main Bioactive Phytochemicals

**FIGURE 2:**
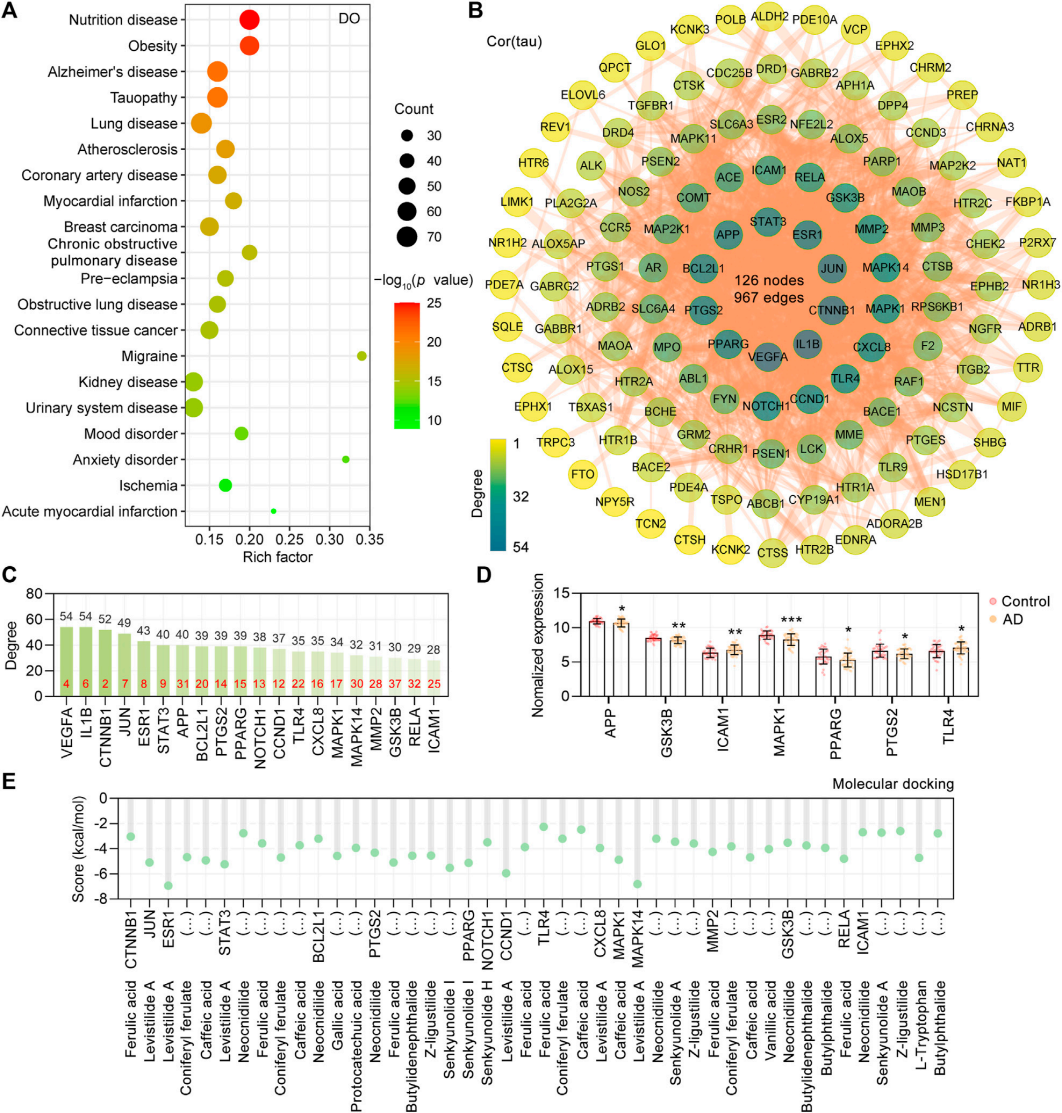
PPI network of CR targets associated with tau pathogenesis. **(A)** Top 20 significantly enriched Disease Ontology (DO) terms are shown as a bubble diagram. DO terms with corrected *p* value <0.05 were considered significantly enriched. The x-axis, rich factor (the ratio of targets in the background terms). Bubble size, the number of genes enriched. Bubble color, *p* value. **(B)** 127 targets related to tau pathogenesis were utilized to construct the PPI network. The color of the node correlates with its degree value. **(C)** The top 20 targets ranked by degree value were identified as the core targets. The red numbers in the bar chart represent the degree value ranking of the core targets in the CR’s 376 target PPI network. **(D)** Differentially expressed core targets of CR related to tau pathogenesis in the temporal cortex of the control (*n* = 39) and AD group (*n* = 52). Data are plotted as individual data points overlaid with mean ± SD. *p < 0.05, **p < 0.01, ***p < 0.001. **(E)** Docking score (kcal/mol) of CR phytochemicals with their corresponding core targets.

Based on the 18 CR phytochemicals and their structures, a total of 376 potential targets were obtained by the SwissTargetPrediction database (Daina et al., 2019). To determine the human disease relevance of these 376 targets, we assessed the functional enrichment by DOSE Bioconductor packages with default parameters for DO (Yu et al., 2015). The DO terms with adjusted *p* values <0.05 were considered significantly enriched, and a total of 555 DO terms were significantly enriched in our study. As shown, the primary enriched DO terms were nutrition disease (DOID: 374), obesity (DOID: 9970), Alzheimer’s disease (DOID: 10652), tauopathy (DOID: 680), and atherosclerosis (DOID: 1936) (Figure 2A). Tauopathy was significantly enriched (adjust *p* value = 7.68E-22), in which 70 targets were involved. Furthermore, the AlzData database (Xu et al., 2018) was used to screen out the tau pathogenesis–associated CR targets, and 70 CR targets were correlated with tau pathology. After all the extracted CR targets were pooled, a total of 127 CR targets were obtained (Supplementary Table S1) and formed a PPI network with 127 nodes, 967 edges, and an average node degree of 15.2 (Figure 2B). Accordingly, 20 targets, namely, VEGFA, IL1B, CTNNB1, JUN, ESR1, STAT3, APP, BCL2L1, PTGS2, PPARG, NOTCH1, CCND1, TLR4, CXCL8, MAPK1, MAPK14, MMP2, GSK3B, RELA, and ICAM1, ranked by degree, were identified as the core targets (Figure 2C). It is noteworthy that we also analyzed the degree value ranking of the above core targets in the PPI network formed by all 376 targets in CR, and the results showed that the core targets are very important in the mechanism of action of CR. Specifically, the core targets CTNNB1, VEGFA, IL1B, JUN, ESR1, and STAT3 ranked in the top 10 in the degree of the PPI network formed by all targets in CR (Figure 2C, red numbers). The AlzData database was used to understand the relationship between these 127 targets and cell types in the human brain. It was also found that 43 targets were localized in neurons, 38 targets in astrocytes, 35 targets in endothelial cells, 34 targets in microglia, and 28 targets in oligodendrocytes, indicating that CR may act on these major types of cells in the brain (Supplementary Figure S2).

**FIGURE 3:**
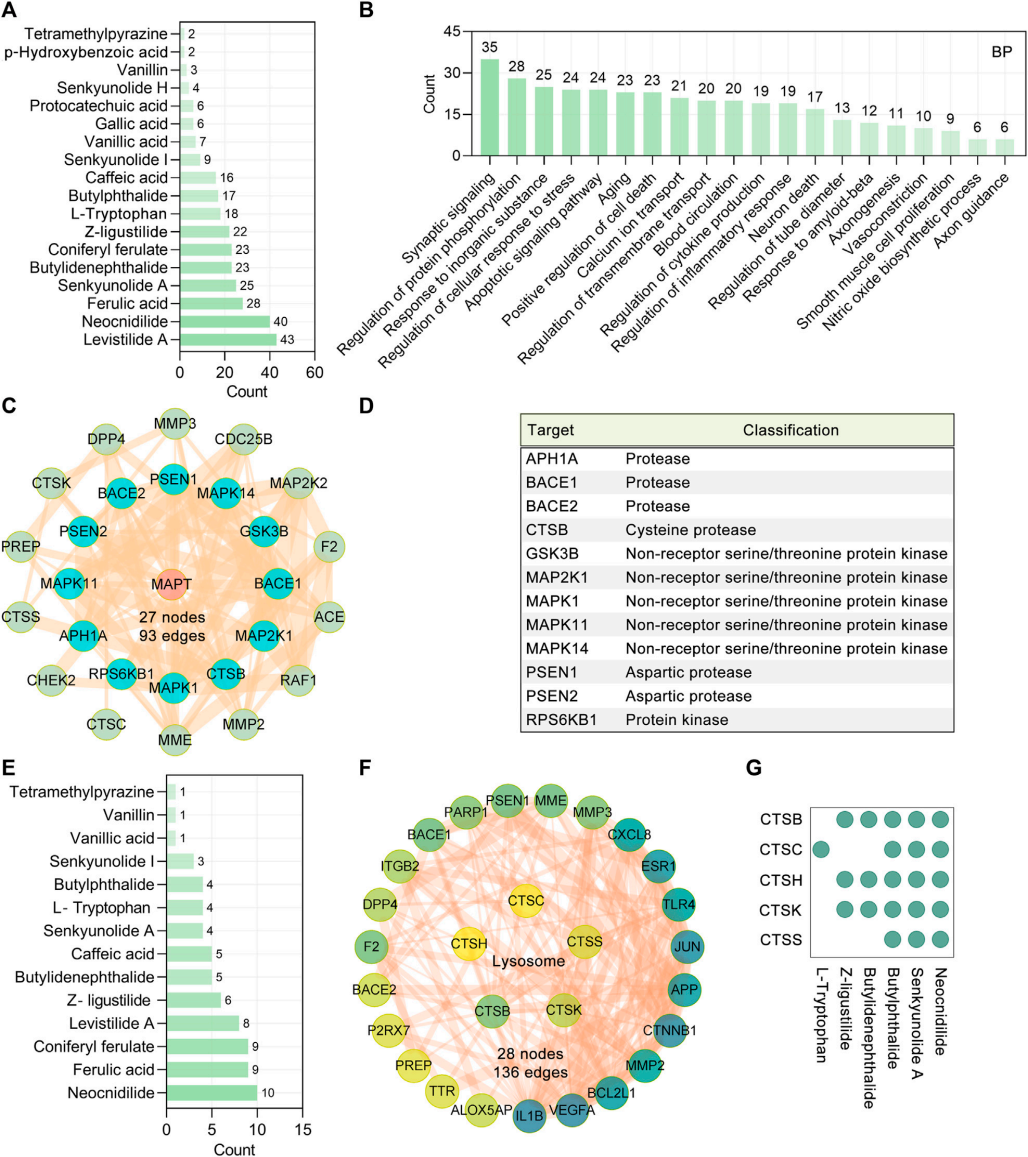
CR targets related to tau aggregation. **(A)** 18 main bioactive phytochemicals of CR and the numbers of their targets associated with tau pathogenesis. The bar diagram displays the numbers of tau pathogenesis–related targets targeted by the phytochemicals. **(B)** GO biological process analysis of CR targets associated with tau pathogenesis. BP terms with a *p* value < 0.05 were considered significant. The bar diagram displaying the numbers of tau pathogenesis–related targets of CR enriched in the different biological processes. **(C)** The PPI network showing the CR targets associated with the modification of tau (encoded by MAPT). Red circle node: MAPT. Cyan and green nodes represent protein modification enzymes directly or indirectly acting on MAPT, respectively. **(D)** Classification of protein modifying enzymes directly acting on tau protein. **(E)** CR phytochemicals involved in tau modification ranked according to the numbers of targets. **(F)** PPI network of CR targets associated with the lysosome pathway (hsa04142). The color of the node correlated with its degree value. **(G)** CR phytochemicals corresponding to targets involved in the lysosome pathway.

Based on the “Differential expression” module of the AlzData database, we analyzed the normalized expression values of the CR core targets related to tau pathogenesis in the healthy control and AD groups in the GEO database (Figure 2D). It was shown that ICAM1 and TLR4 were significantly upregulated, and APP, GSK3B, MAPK1, PPARG, and PTGS2 were significantly downregulated in the AD temporal cortex (Figure 2D). To investigate the possibility of the core targets binding to their corresponding CR phytochemicals, we performed the molecular docking analysis. The results showed that all the core targets were well combined with the corresponding phytochemicals (Figure 2E). Among them, levistilide A showed the highest binding energy with ESR1 (score value −6.94 kcal/mol), MAPK14 (score value −6.81 kcal/mol), CCND1 (score value −5.94 kcal/mol), and STAT3 (score value −5.24 kcal/mol). By studying the phytochemicals corresponding to the 127 tau pathogenesis–related CR targets, we found that levistilide A (43 targets), neocnidilide (40 targets), ferulic acid (28 targets), senkyunolide A (25 targets), butylidenephthalide (23 targets), coniferyl ferulate (23 targets), and Z-ligustilide (22 targets) have the highest number of targets (Figure 3A). The chemical structures of the CR phytochemicals with the top 10 anti-tau pathogenesis targets are presented in Supplementary Figure S3. All 18 main bioactive phytochemicals of CR are associated with tau pathogenesis, and most of them correspond to multiple molecules associated with tau pathogenesis (Figure 3A).

### Major Effects of CR Main Bioactive Phytochemicals on Tau Aggregation

In principle, the synthesis, folding, posttranslational processing, and degradation affect the intracellular homeostasis of tau. Inappropriate posttranslational modifications, such as (but not limited to) phosphorylation, glycosylation, acetylation, ubiquitination, glycation, SUMOylation, methylation, and nitration, have long been recognized as contributing to the aggregation and toxicity of tau. Among all posttranslational modifications of tau, phosphorylation is the most studied. It is widely believed that tau undergoes hyperphosphorylation before accumulating into AD-associated aggregates. GO functional enrichment showed that in the regulation of protein phosphorylation (GO: 0001934), there are 28 targets involved (Figure 3B). In this research, the protein functional classification revealed that 29 of 127 tau pathogenesis–associated targets were protein-modifying enzymes (PC00260). To screen the protein-modifying enzymes that can directly interact with tau, we further used the STRING database (version 11.5) (Szklarczyk et al., 2019) to construct a PPI network. It was found that tau (encoded by MAPT) hyperphosphorylation involved protein kinases GSK3B, MAP2K1, MAPK1, MAPK11, MAPK14, and RPS6KB1 were the major tau modification-related targets of CR (Figures 3C,D). The present data did not show any effects of CR on other posttranslational modifications of tau. The possible regulators of tau hyperphosphorylation were suggested according to the number of related targets (Figure 3E). Neocnidilide, ferulic acid, coniferyl ferulate, levistilide A, and Z-ligustilide were the top five tau hyperphosphorylation regulators. In addition, it was shown that the amyloid precursor protein (APP) sequential cleavage-associated proteases APH1A, BACE1, BACE2, CTSB, PSEN1, and PSEN2 were also the targets of CR (Figure 3D).

Insufficient degradation promotes tau aggregation. There are two main pathways for tau degradation, the ubiquitin–proteasome pathway and lysosomal proteolysis (Wang et al., 2009). The KEGG enrichment analysis revealed that 127 CR targets were significantly enriched in the lysosome (hsa04142, *p* = 2.71E-04, CTSB, CTSC, CTSH, CTSK, and CTSS), but not proteasome pathway (Figure 3F). Cathepsins (CTSs) are mainly lysosomal hydrolases and comprise 15 proteolytic enzymes, which are classified into three distinct groups, namely, serine (CTSA and CTSG), cysteine (CTSB, CTSC, CTSH, CTSF, CTSL, CTSK, CTSO, CTSS, CTSV, CTSX, and CTSW), and aspartate (CTSD and CTSE). There were 23 tau pathogenesis–associated targets having direct interactions with CTSB, CTSC, CTSH, CTSK, and CTSS (Figure 3F), indicating that CR may promote tau degradation through the lysosomal pathway. The possible regulators of the tau lysosomal degradation are suggested to be butylidenephthalide, butylphthalide, L-tryptophan, neocnidilide, senkyunolide A, and Z-ligustilide (Figure 3G).

### Major Effects of CR Main Bioactive Phytochemicals on Tau-Mediated Toxicities

**FIGURE 4:**
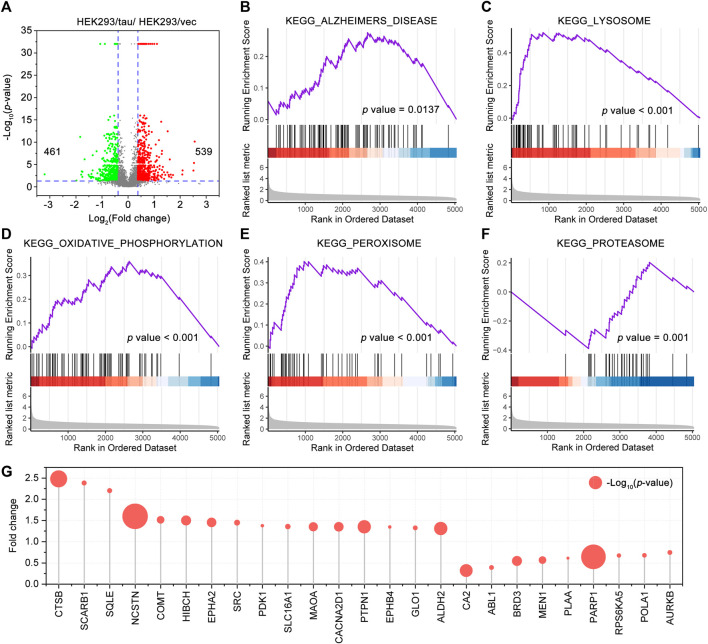
Gene set enrichment analysis (GSEA) of the proteins in HEK293/tau cells (stable overexpressing human wild-type full-length tau) and HEK293/vec cells. **(A)** Volcano plot showing the differentially expressed proteins, which were as differentially expressed when fold change >1.3 and *p* < 0.05, between HEK293/tau cells and HEK293/vec cells. GSEA of the proteins identified by proteomics. KEGG_ALZHEIMERS DISEASE **(B)**, the KEGG LYSOSOME **(C)**, KEGG OXIDATIVE PHOSPHORYLATION **(D)**, KEGG PEROXISOME **(E)**, and KEGG PROTEASOME **(F)** were significantly enriched. **(G)** The common proteins of the differentially expressed protein data set (HEK293/tau vs HEK293/vec cells) and CR target data set. The size of each bubble point represents the corresponding *p* value.

Tau is normally enriched on the microtubules within the neuron. Tau aggregation and mislocalization alter Ca^2+^ homeostasis impair the intracellular transport such as axonal transport, induce synaptic loss and dysfunction, impair the structures, trafficking, and functions of the organelles, and lead to neuron death, underlying the tau-mediated toxicities (Bok et al., 2021; Zhang et al., 2021). To provide an insight into tau-mediated toxicities, we conducted a proteomic profiling of the HEK293/tau cells and HEK293/vec cells (*n* = 3/group). A total of 5,044 proteins were identified, of which 539 proteins were upregulated and 461 proteins were downregulated proteins in the HEK293/tau cells (Figure 4A). By the GSEA analysis with higher repeatability and accuracy than the KEGG and GO analysis, a total of 41 KEGG pathways were significantly enriched (*p* < 0.05). A significant enrichment in KEGG_ALZHEIMERS_DISEASE (NES = 1.64, *p* = 0.0137) (Figure 4B) was shown. Furthermore, the KEGG_LYSOSOME (NES = 2.97, *p* < 0.001), KEGG_OXIDATIVE_PHOSPHORYLATION (NES = 2.07, *p* < 0.001), KEGG_PEROXISOME (NES = 2.09, *p* < 0.001), and KEGG_PROTEASOME (NES = −2.89, *p* = 0.001) were also significantly enriched (Figures 4C–F). Among these pathways, the number of proteins involved in the KEGG_ALZHEIMERS_DISEASE was the largest (87 proteins), followed by KEGG_OXIDATIVE_PHOSPHORYLATION (72 proteins), and KEGG_LYSOSOME (67 proteins). We also used Venny 2.1 to sort the common proteins of the HEK293/tau differentially expressed protein data set and CR target data set, and a total of 25 common targets were obtained, namely, CTSB, SCARB1, SQLE, NCSTN, COMT, HIBCH, EPHA2, SRC, PDK1, SLC16A1, CA2, ABL1, BRD3, MEN1, PLAA, PARP1, RPS6KA5, POLA1, and AURKB (Figure 4G). Among these targets, ALDH2, COMT, EPHA2, HIBCH, MAOA, and PARP1 were also differentially expressed in the temporal cortex of the AD patients and had the same change trends (Supplementary Figure S4).

**FIGURE 5:**
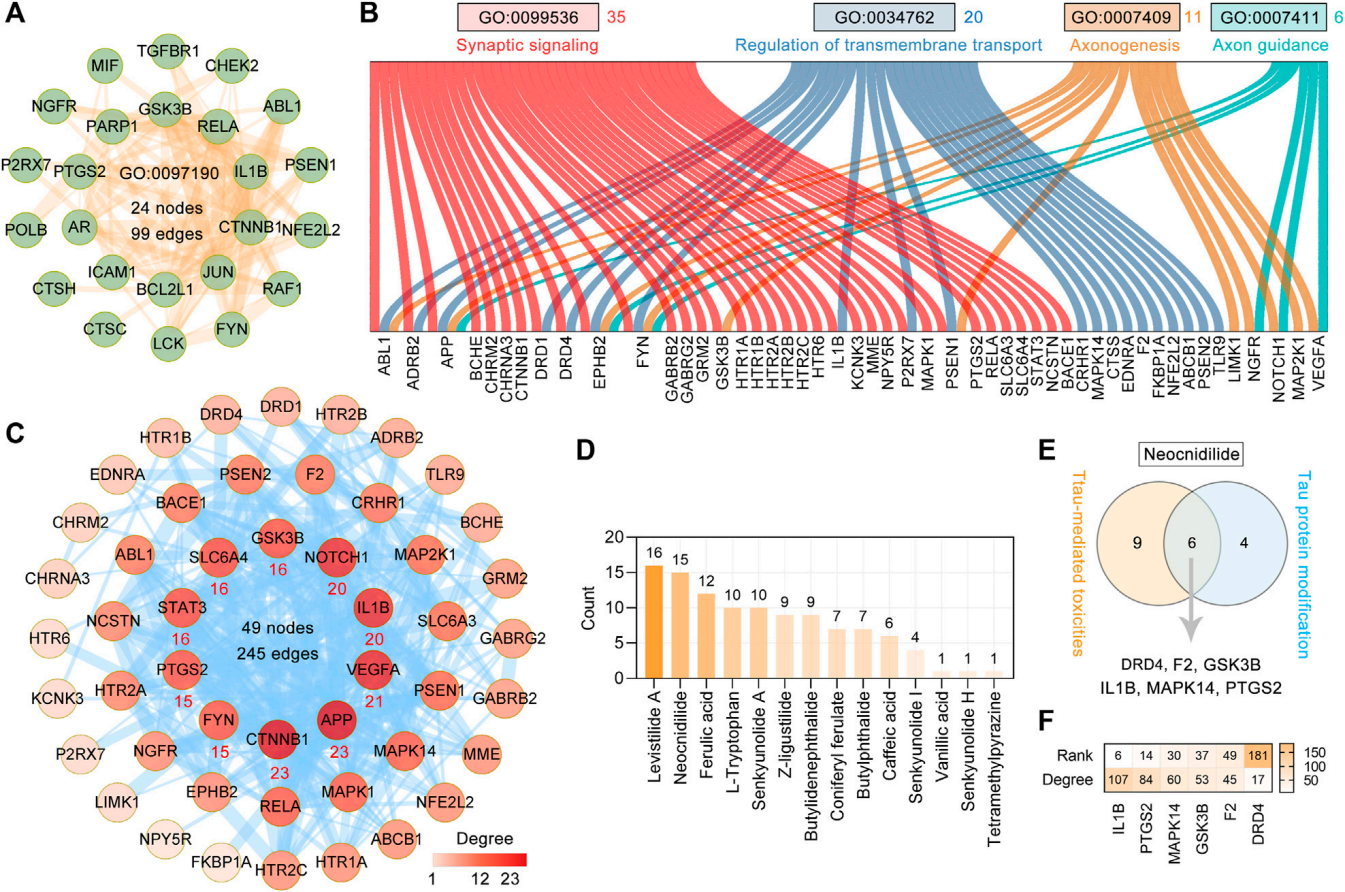
Major effects of CR main bioactive phytochemicals on tau-mediated toxicities. **(A)** The PPI network of 24 CR targets involved in the apoptotic signaling pathway (GO: 0097190). **(B)** Sankey diagram of CR targets involved in synaptic signaling (GO: 0099536), regulation of transmembrane transport (GO: 0034762), axonogenesis (GO: 0007409), and axon guidance (GO: 0007411). **(C)** The PPI network was constructed for 50 targets of CR associated with tau-mediated toxicities. The darker the node color, the higher degree it represents. The thickness of the edge denotes the combined score. The red number below the node represents the top 10 core targets ranked by degree. **(D)** CR phytochemicals involved in tau-mediated toxicities ranked according to the numbers of their targets. **(E)** Neocnidilide acts in both tau modification and tau-mediated toxicities *via* DRD4, F2, GSK3B, IL1B, MAPK14, and PTGS2. **(F)** In the PPI network of 376 CR targets, the degree value ranking of the six targets associated with **(E)**.

To explore the effect of CR on tau-mediated toxicities, we performed functional enrichment of 127 targets by GO BP terms. The top 20 enriched GO BP terms ranked by the count are shown in Supplementary Table S2 and Figure 3B. The primary enriched BP terms were synaptic signaling (GO: 0099536), regulation of protein phosphorylation (GO: 0001934), apoptotic signaling pathway (GO: 0097190), regulation of transmembrane transport (GO: 0034762), neuron death (GO: 0070997), axonogenesis (GO: 0007409), and axon guidance (GO: 0007411). In the apoptotic signaling pathway (GO: 0097190), 24 targets are involved and formed a complex PPI network with 24 nodes and 99 edges (Figure 5A). Based on Figure 3B and Figure 5B, 35 targets are involved in synaptic signaling (GO: 0099536), 20 in regulation of transmembrane transport (GO: 0034762), 11 in axonogenesis (GO: 0007409), and six in axon guidance (GO: 0007411). In these targets and the STRING database (version 11.5)–based PPI network (Figure 4B), the top 10 targets are CTNNB1, APP, VEGFA, IL1B, NOTCH1, GSK3B, SLC6A4, STAT3, PTGS2, and FYN (Figure 5C). Further analysis showed that the main contributors to these targets are levistilide A (16 targets), neocnidilide (15 targets), ferulic acid (12 targets), L-tryptophan (10 targets), senkyunolide A (10 targets), Z-ligustilide (9 targets), and butylidenephthalide (9 targets) (Figure 5D). In addition, six targets of neocnidilide (DRD4, F2, GSK3B, IL1B, MAPK14, and PTGS2) act both in tau aggregation and tau-mediated toxicities (Figure 5E). The degree ranking of these targets in the PPI network formed by 376 targets of CR is also displayed in Figure 5F. All these data suggest that the main bioactive phytochemicals of CR antagonize tau-mediated impairments of intracellular transport, axon and synaptic damages, and neuron death (especially apoptosis).

## Discussion

**FIGURE 6:**
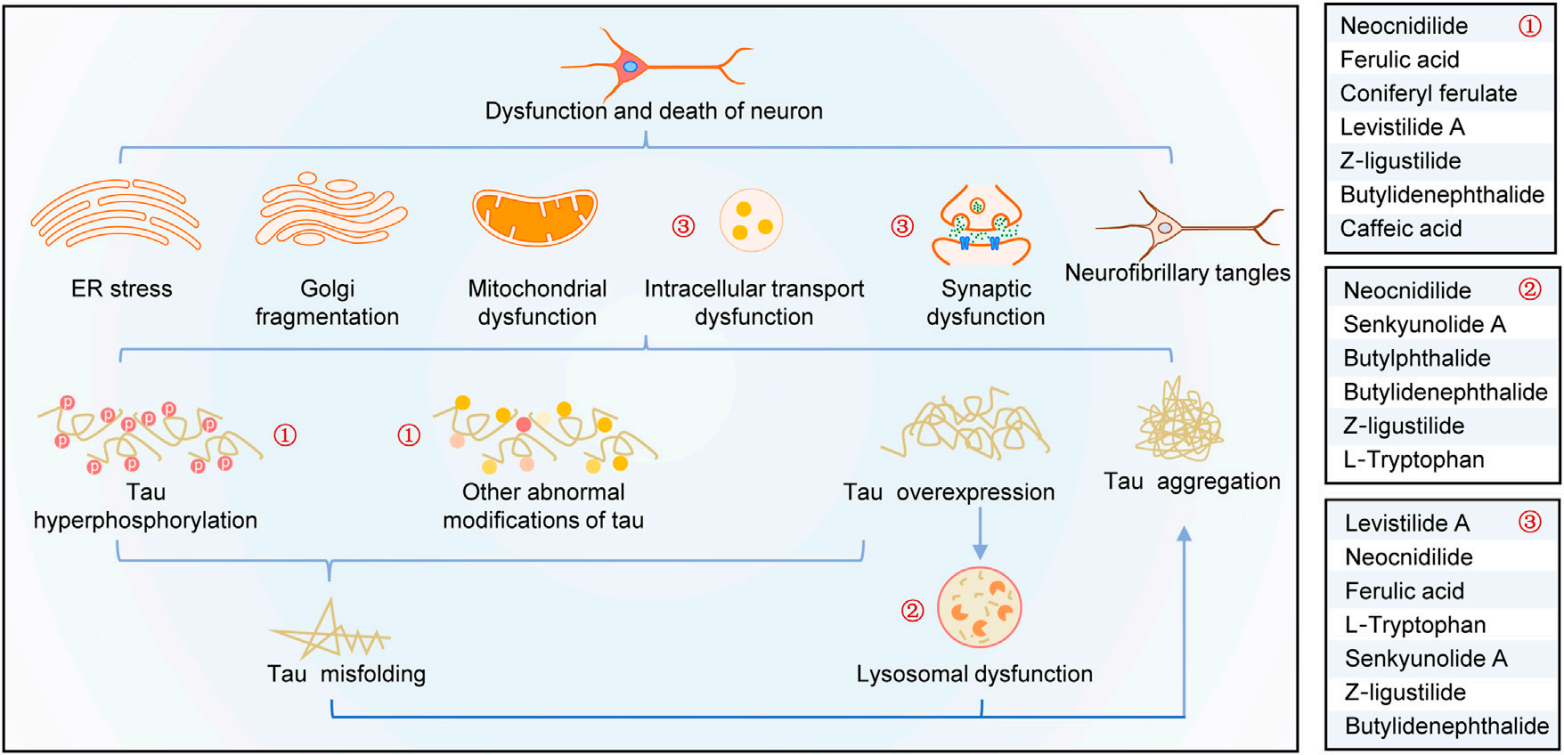
Schematic diagram of the mechanisms of CR treatment of AD based on tau pathogenesis. CR phytochemicals related to the modification (①), degradation (②), and toxicities (③) of tau.

A summary of this study is shown in Figure 6. Overexpression, abnormal modifications, and degradation obstruction make tau proteins accumulate in the neurons, resulting in a series of neurotoxic effects, such as organelle dysfunctions, axon damages, synaptic loss and dysfunction, intracellular transport disorders, and neuronal death. By analyzing tau pathogenesis–associated targets of CR bioactive phytochemicals and their effects on tau aggregation and tau-mediated toxicities, it is shown that CR targets tau hyperphosphorylation, promotes tau degradation through the lysosomal pathway, and antagonizes tau-mediated impairments of intracellular transport, axon and synaptic damages, and neuron death (especially apoptosis). As shown in Figure 6, the major bioactive phytochemicals of CR targeting tau pathogenesis include neocnidilide, ferulic acid, ferulic acid, Z-ligustilide, and butylidenephthalide.

There are many challenges in network pharmacology studies. First, the chemical composition of a traditional Chinese medicine compound is the material basis of its efficacy, but it is worth noting that the traditional Chinese medicine compound acts as a whole, and its chemical composition is not a simple addition of the effects of a single herb. Therefore, it is very unconvincing to add the chemical constituents of single herbs contained in a traditional Chinese medicine compound using public databases as compound ingredients. Second, current research generally uses the oral bioavailability and drug likeness properties of chemical components to screen bioactive phytochemicals and rarely considers the dose–effect relationship of traditional Chinese medicine phytochemicals. Third, the use of public databases to obtain disease targets ignores the pathophysiological process of disease development and increases subjectivity. Fourth, the toxicity of phytochemicals, such as hepatotoxicity, is not considered. Fifth, the existence of non-intersection drug targets is ignored after the intersection of drugs and disease targets, and the importance of intersection targets for drugs and diseases is not proved. Sixth, current research usually only considers the target proteins corresponding to phytochemicals and rarely considers the combination of phytochemicals and other biological function molecules.

Compared with the previous studies, this study has the following improvements. First, we used a novel strategy to uncover the key phytochemicals of CR associated with tau pathogenesis. This avoids the subjectivity of directly focusing on the symptoms and end-pathological changes of AD in obtaining the targets using public databases. Second, the present study is based on the active CR phytochemicals determined by UHPLC MS/MS studies, namely, aromatic acids, phthalides, and TMP (Wang et al., 2020; Yin et al., 2019). It is noteworthy that the distribution of the main bioactive phytochemicals of CR focused in this study is highly specific, such as butylidenephthalide, neocnidilide, senkyunolide A, and Z-ligustilide, which are only present in 3, 6, 10, and 13 herbs, respectively, including CR (data from HERB, http://herb.ac.cn/) (Fang et al., 2021). In stark contrast, numerous network pharmacology studies have identified quercetin (related to 341 herbs), kaempferol (related to 248 herbs), luteolin (related to 181 herbs), and β-sitosterol (related to 567 herbs), among others, as candidate phytochemicals for different diseases (Wang Y. et al., 2021; Zhou et al., 2021). These widely distributed phytochemicals are highly unlikely to be the herb-specific active ingredients. Third, we also assessed the contents of the identified CR bioactive phytochemicals and the toxicological parameters and used the human high-throughput omics data to validate the targets of CR related to tau pathogenesis in the AD brain. Undoubtedly, this study has some limitations. First, the results of this study need to be further validated, especially in AD transgenic animal models and related cell models. Second, we also found that RO5-violating CR phytochemicals (chlorogenic acid, cryptochlorogenic acid, and 3,5-O-dicaffeoylquinic acid) have some neuroprotective effects (Kwon et al., 2010; Heitman and Ingram, 2017; Ji et al., 2021). This suggested that overemphasizing RO5 has the dangerous potential to overfilter compounds that may otherwise be good drug candidates and this inevitably results in reduced productivity for discovering new drugs. Notably, the above three RO5-violating CR phytochemicals exhibited the worst BBB penetration (with the smallest BBB parameter).

TMP, the most studied bioactive phytochemical of CR, is widely used to treat brain impairments caused by infarction/reperfusion (Liao et al., 2004; Fan et al., 2006). However, we found that only two of TMP targets (VEGFA and TBXAS1) were associated with tau pathogenesis, suggesting it might have no therapeutic significance for tau disease. Some studies have found that reduced levels of VEGFA protein in the serum and cerebrospinal fluid are associated with an increased risk of AD and cognitive decline (Hohman et al., 2015; Moore et al., 2020). A previous study indicated that after oral administration of CR, 13 phytochemicals were absorbed into the rat plasma in prototype form (Zuo et al., 2011). Among them, the ferulic acid, butylidenephthalide, levistolide A, neocnidilide, senkyunolide A, senkyunolide I, and Z-ligustilide analyzed in this study are included. An isobenzofurans organic compound of CR, neocnidilide, has a variety of biological activities such as insecticidal activity (Tsukamoto et al., 2005) and anti-fungal activity (Marongiu et al., 2013). Here, we showed that it is important in treating tau-related diseases, as it acts in both tau modification and tau-mediated toxicities *via* its targets DRD4, F2, GSK3B, IL1B, MAPK14, and PTGS2. As one of these six key targets of neocnidilide, GSK3β is a widely expressed serine threonine kinase in the brain, and its expression increases in aging and AD (Lee et al., 2006). GSK3β activation has been directly linked to neurodegeneration by promoting tau hyperphosphorylation, memory consolidation, neurogenesis, synaptic plasticity, long-term potentiation, and also neuroinflammation (Lauretti et al., 2020). Therefore, it is considered to be an important target for AD prevention and treatment. Levistilide A, a bioactive phytochemical of CR with anti-inflammatory activities and potential P-gp modulation, is currently being used for treating cancer (Guo et al., 2018). In 6-month-old APP/PS1 transgenic mice, 4 months of intraperitoneal injection with levistilide A (2 mg/kg/day) rescued memory deficits *via* decreasing Aβ aggregation, tau hyperphosphorylation, and synapse loss (Qu et al., 2021). Senkyunolide A, a standard compound for the quality evaluation of CR, has the vasorelaxation activity in contractions to various contractile agents in rat isolated aorta (Chan et al., 2007). Furthermore, recent studies have also indicated that senkyunolide A shows anti-bacterial (Kim et al., 2020) and immunomodulatory activities by inhibiting activating protein-1 (AP-1) and NF-κB (Lei et al., 2019). Z-ligustilide exerts various pharmacological properties, such as neuroprotection, anti-inflammation, and anti-oxidization (Xie et al., 2020). *In vitro* studies suggest that Z-ligustilide protects against Aβ fibrils–induced neurotoxicity by inhibiting p38 and activating the PI3K/Akt signaling pathway (Xu et al., 2016). Therefore, more attention should be paid to these key phytochemicals of CR in the treatment of tau-related diseases in the future.

The tau protein has 85 putative phosphorylation sites (Hanger et al., 2009), which are regulated by a series of protein kinases and phosphatases (Carroll et al., 2021). Hyperphosphorylation is the most documented posttranslational modification in tau pathogenesis. Tau undergoes hyperphosphorylation before aggregating. In this study, we showed that GSK3β is a target of neocnidilide, butylidenephthalide, and butylphthalide. GSK3 regulates more than 40 putative tau phosphorylation sites, of which at least 29 are hyperphosphorylated in the AD brain (Hanger et al., 2009). Butylidenephthalide significantly reduced activated GSK3β–induced tau hyperphosphorylation at serine 396 in neurons (Chang et al., 2015). Furthermore, GSK3β is implicated in the regulation of neuronal apoptosis. Transgenic mice overexpressing GSK3β exhibit tau hyperphosphorylation, disrupted microtubules, and neuronal apoptosis (Lucas et al., 2001). The core target CTNNB1 encodes β-catenin, which is an important downstream of GSK3β. The Wnt/β-catenin signaling regulates multiple distinct pathways in the pathogenesis of AD such as synaptic plasticity, neuronal survival, neurogenesis, BBB integrity, tau phosphorylation, and Aβ production (Jia et al., 2019). Protein phosphatases such as the major phosphatase dephosphorylating tau, protein phosphatase 2A (PP2A), were not shown to be regulated by CR in this study. Notably, ERα (encoded by ESR1) localizes to neurofibrillary tangles in the AD brain and has increased interaction with tau, which likely resulted in a large amount of ERα being sequestered in the paired helical filaments and neuritic tau pathology in the AD brain (Wang C. et al., 2016). The clearance of physiological and pathological tau is mainly mediated by the ubiquitin proteasome system and autophagy/lysosomal degradation pathway. In AD patients, the neuronal lysosome system was dysregulated (Sundelof et al., 2010; Wang X. M. et al., 2021; Liu et al., 2021; Hamano et al., 2021). The results of this study show that CR promotes the degradation of pathological tau mainly through the lysosome system rather than the proteasome system. Ca^2+^ homeostasis imbalance, impairments of the intracellular transport such as axonal transport, synaptic loss and dysfunction, impairments of the structures, trafficking and functions of organelles, and neuron death are reported as tau-mediated toxicities (Bok et al., 2021; Zhang et al., 2021). This study shows that the main bioactive phytochemicals of CR antagonize tau-mediated impairments of intracellular transport, axon and synaptic damages, and neuron death.

## Conclusion

In this study, we present a novel network pharmacology approach based on disease-specific pathophysiological processes and screen out a total of 18 bioactive phytochemicals in CR that corresponded to 127 targets correlated with tau pathogenesis. VEGFA, IL1B, CTNNB1, JUN, ESR1, STAT3, APP, BCL2L1, PTGS2, and PPARG were identified as core targets that play an important role in the treatment of tau pathogenesis. Neocnidilide, ferulic acid, coniferyl ferulate, levistilide A, Z-ligustilide, butylidenephthalide, and caffeic acid were identified as the key phytochemicals regulating tau protein modification, while neocnidilide, senkyunolide A, butylphthalide, butylidenephthalide, Z-ligustilide, and L-tryptophan were identified as the key phytochemicals regulating tau protein degradation, and levistilide A, neocnidilide, ferulic acid, L-tryptophan, senkyunolide A, Z-ligustilide, and butylidenephthalide were identified as the key phytochemicals regulating tau-mediated toxicities. Taken together, this study screened a variety of candidate drugs strongly associated with tau pathogenesis and provides a basis for the use of CR phytochemicals as a therapeutic for tau pathology–related neurodegenerative diseases.

## Data Availability

The data sets presented in this study can be found in online repositories. The names of the repository/repositories and accession number(s) can be found in the article/Supplementary Material.

## Abbreviations

AD, Alzheimer’s disease; APP, amyloid precursor protein; Aβ, amyloid-β; BBB, blood–brain barrier; BP, biological process; CC, cellular component; CR, *Chuanxiong Rhizoma*; CTSs, cathepsins; DO, Disease Ontology; GEO, Gene Expression Omnibus; GO, gene ontology; GSEA, gene set enrichment analysis; Hacc, hydrogen bond acceptors; Hdon, hydrogen bond donors; iTRAQ, isobaric tags for relative and absolute quantification; KEGG, Kyoto Encyclopedia of Genes and Genomes; LogP, lipid–water partition coefficient; MF, molecular function; MW, molecule weight; NES, normalized enrichment score; NFTs, neurofibrillary tangles; NF-κB, nuclear factor κB; NMDA, N-methyl-D-aspartic acid; PP2A, protein phosphatase 2A; PPI, protein–protein interaction; Rbon, rotatable bonds; RO5, Lipinski’s rule of five; SAscore, synthetic accessibility score; TCMSP, Traditional Chinese Medicine Systems Pharmacology Database And Analysis Platform; TMP, tetramethylpyrazine; TNFα, tumor necrosis factor α; UHPLC-MS/MS, ultra-HPLC with triple quadrupole mass spectrometry.

## References

[B1] Banerjee P. Eckert A. O. Schrey A. K. Preissner R. (2018). ProTox-II: a Webserver for the Prediction of Toxicity of Chemicals. Nucleic Acids Res. 46 (W1), W257–W263. 10.1093/nar/gky318 29718510 PMC6031011

[B2] Bok E. Leem E. Lee B. R. Lee J. M. Yoo C. J. Lee E. M. (2021). Role of the Lipid Membrane and Membrane Proteins in Tau Pathology. Front. Cel. Dev. Biol. 9, 653815. 10.3389/fcell.2021.653815 PMC8119898 33996814

[B3] Braak H. Braak E. (1991). Neuropathological Stageing of Alzheimer-Related Changes. Acta Neuropathol. 82 (4), 239–259. 10.1007/BF00308809 1759558

[B4] Buerger K. Ewers M. Pirttilä T. Zinkowski R. Alafuzoff I. Teipel S. J. (2006). CSF Phosphorylated Tau Protein Correlates with Neocortical Neurofibrillary Pathology in Alzheimer's Disease. Brain 129 (Pt 11), 3035–3041. 10.1093/brain/awl269 17012293

[B5] Cai X. Chen Z. Pan X. Xia L. Chen P. Yang Y. (2014). Inhibition of Angiogenesis, Fibrosis and Thrombosis by Tetramethylpyrazine: Mechanisms Contributing to the SDF-1/CXCR4 Axis. PLoS One 9 (2), e88176. 10.1371/journal.pone.0088176 24505417 PMC3914919

[B6] Carroll T. Guha S. Nehrke K. Johnson G. V. W. (2021). Tau Post-Translational Modifications: Potentiators of Selective Vulnerability in Sporadic Alzheimer's Disease. Biology (Basel) 10 (10), 1047. 10.3390/biology10101047 34681146 PMC8533264

[B7] Chan S. S. Cheng T. Y. Lin G. (2007). Relaxation Effects of Ligustilide and Senkyunolide A, Two Main Constituents of Ligusticum Chuanxiong, in Rat Isolated Aorta. J. Ethnopharmacol. 111 (3), 677–680. 10.1016/j.jep.2006.12.018 17222996

[B8] Chang C. Y. Chen S. M. Lu H. E. Lai S. M. Lai P. S. Shen P. W. (2015). N-butylidenephthalide Attenuates Alzheimer's Disease-like Cytopathy in Down Syndrome Induced Pluripotent Stem Cell-Derived Neurons. Sci. Rep. 5, 8744. 10.1038/srep08744 25735452 PMC4348654

[B9] Chen Z. Zhang C. Gao F. Fu Q. Fu C. He Y. (2018). A Systematic Review on the Rhizome of Ligusticum Chuanxiong Hort. (Chuanxiong). Food Chem. Toxicol. 119, 309–325. 10.1016/j.fct.2018.02.050 29486278

[B10] Daina A. Michielin O. Zoete V. (2017). SwissADME: A Free Web Tool to Evaluate Pharmacokinetics, Drug-Likeness and Medicinal Chemistry Friendliness of Small Molecules. Sci. Rep. 7, 42717. 10.1038/srep42717 28256516 PMC5335600

[B11] Daina A. Michielin O. Zoete V. (2019). SwissTargetPrediction: Updated Data and New Features for Efficient Prediction of Protein Targets of Small Molecules. Nucleic Acids Res. 47 (W1), W357–W364. 10.1093/nar/gkz382 31106366 PMC6602486

[B12] Donkor P. O. Chen Y. Ding L. Qiu F. (2016). Locally and Traditionally Used Ligusticum Species - A Review of Their Phytochemistry, Pharmacology and Pharmacokinetics. J. Ethnopharmacol. 194, 530–548. 10.1016/j.jep.2016.10.012 27729283

[B13] Dubois B. Feldman H. H. Jacova C. Hampel H. Molinuevo J. L. Blennow K. (2014). Advancing Research Diagnostic Criteria for Alzheimer's Disease: the IWG-2 Criteria. Lancet Neurol. 13 (6), 614–629. 10.1016/S1474-4422(14)70090-0 24849862

[B14] Ertl P. Schuffenhauer A. (2009). Estimation of Synthetic Accessibility Score of Drug-like Molecules Based on Molecular Complexity and Fragment Contributions. J. Cheminform. 1 (1), 8. 10.1186/1758-2946-1-8 20298526 PMC3225829

[B15] Fan L. H. Wang K. Z. Cheng B. Wang C. S. Dang X. Q. (2006). Anti-apoptotic and Neuroprotective Effects of Tetramethylpyrazine Following Spinal Cord Ischemia in Rabbits. BMC Neurosci. 7, 48. 10.1186/1471-2202-7-48 16774675 PMC1534051

[B16] Fang S. Dong L. Liu L. Guo J. Zhao L. Zhang J. (2021). HERB: A High-Throughput Experiment- and Reference-Guided Database of Traditional Chinese Medicine. Nucleic Acids Res. 49 (D1), D1197–D1206. 10.1093/nar/gkaa1063 33264402 PMC7779036

[B17] Fang Y. Y. Zeng P. Qu N. Ning L. N. Chu J. Zhang T. (2018). Evidence of Altered Depression and Dementia-Related Proteins in the Brains of Young Rats after Ovariectomy. J. Neurochem. 146 (6), 703–721. 10.1111/jnc.14537 29939407

[B18] Guo H. Sun L. Ling S. Xu J. W. (2018). Levistilide A Ameliorates NLRP3 Expression Involving the Syk-p38/JNK Pathway and Peripheral Obliterans in Rats. Mediators Inflamm. 2018, 7304096. 10.1155/2018/7304096 30158835 PMC6109531

[B19] Hamano T. Enomoto S. Shirafuji N. Ikawa M. Yamamura O. Yen S.-H. (2021). Autophagy and Tau Protein. Ijms 22 (14), 7475. 10.3390/ijms22147475 34299093 PMC8303176

[B20] Hampel H. Bürger K. Pruessner J. C. Zinkowski R. Debernardis J. Kerkman D. (2005). Correlation of Cerebrospinal Fluid Levels of Tau Protein Phosphorylated at Threonine 231 with Rates of Hippocampal Atrophy in Alzheimer Disease. Arch. Neurol. 62 (5), 770–773. 10.1001/archneur.62.5.770 15883264

[B21] Han D. Yang X. Shi J. Tian J. (2014). Analysis on Therapies and Medications in Randomized Controlled Trials of TCM for Dementia. J. Tradit. Chin. Med. 55 (12), 1051–1054.

[B22] Hanger D. P. Anderton B. H. Noble W. (2009). Tau Phosphorylation: The Therapeutic Challenge for Neurodegenerative Disease. Trends Mol. Med. 15 (3), 112–119. 10.1016/j.molmed.2009.01.003 19246243

[B23] Heitman E. Ingram D. K. (2017). Cognitive and Neuroprotective Effects of Chlorogenic Acid. Nutr. Neurosci. 20 (1), 32–39. 10.1179/1476830514Y.0000000146 25130715

[B24] Hinz F. I. Geschwind D. H. (2017). Molecular Genetics of Neurodegenerative Dementias. Cold Spring Harb. Perspect. Biol. 9 (4). 10.1101/cshperspect.a023705 PMC5378052 27940516

[B25] Hohman T. J. Bell S. P. Jefferson A. L. (2015). The Role of Vascular Endothelial Growth Factor in Neurodegeneration and Cognitive Decline: Exploring Interactions with Biomarkers of Alzheimer Disease. JAMA Neurol. 72 (5), 520–529. 10.1001/jamaneurol.2014.4761 25751166 PMC4428948

[B26] Huang X. Yang J. Huang X. Zhang Z. Liu J. Zou L. (2021). Tetramethylpyrazine Improves Cognitive Impairment and Modifies the Hippocampal Proteome in Two Mouse Models of Alzheimer's Disease. Front. Cel. Dev. Biol. 9, 632843. 10.3389/fcell.2021.632843 PMC8005584 33791294

[B27] Ji X. Wang B. Paudel Y. N. Li Z. Zhang S. Mou L. (2021). Protective Effect of Chlorogenic Acid and its Analogues on Lead-Induced Developmental Neurotoxicity through Modulating Oxidative Stress and Autophagy. Front. Mol. Biosci. 8, 655549. 10.3389/fmolb.2021.655549 34179077 PMC8226318

[B28] Jia L. Piña-Crespo J. Li Y. (2019). Restoring Wnt/β-Catenin Signaling Is a Promising Therapeutic Strategy for Alzheimer's Disease. Mol. Brain 12 (1), 104. 10.1186/s13041-019-0525-5 31801553 PMC6894260

[B29] Kim S. Thiessen P. A. Cheng T. Yu B. Shoemaker B. A. Wang J. (2016). Literature Information in PubChem: Associations between PubChem Records and Scientific Articles. J. Cheminform 8, 32. 10.1186/s13321-016-0142-6 27293485 PMC4901473

[B30] Kim T. Y. Kwon H. C. Lee S. Y. Lee C. M. Lee K. S. Lee K. K. (2020). Antibacterial Activity of Senkyunolide A Isolated from Cnidium Officinale Extract. J. Cosmet. Sci. 71 (6), 377–383. 33413782

[B31] Kobayashi S. Mimura Y. Notoya K. Kimura I. Kimura M. (1992). Antiproliferative Effects of the Traditional Chinese Medicine Shimotsu-To, its Component Cnidium Rhizome and Derived Compounds on Primary Cultures of Mouse Aorta Smooth Muscle Cells. Jpn. J. Pharmacol. 60 (4), 397–401. 10.1254/jjp.60.397 1287277

[B32] Kuang X. Du J. R. Chen Y. S. Wang J. Wang Y. N. (2009). Protective Effect of Z-Ligustilide against Amyloid Beta-Induced Neurotoxicity Is Associated with Decreased Pro-inflammatory Markers in Rat Brains. Pharmacol. Biochem. Behav. 92 (4), 635–641. 10.1016/j.pbb.2009.03.007 19324070

[B33] Kwon S. H. Lee H. K. Kim J. A. Hong S. I. Kim H. C. Jo T. H. (2010). Neuroprotective Effects of Chlorogenic Acid on Scopolamine-Induced Amnesia via Anti-acetylcholinesterase and Anti-oxidative Activities in Mice. Eur. J. Pharmacol. 649 (1-3), 210–217. 10.1016/j.ejphar.2010.09.001 20854806

[B34] Lalli G. Schott J. M. Hardy J. De Strooper B. (2021). Aducanumab: a New Phase in Therapeutic Development for Alzheimer's Disease? EMBO Mol. Med. 13 (8), e14781. 10.15252/emmm.202114781 34338436 PMC8350893

[B35] Lauretti E. Dincer O. Praticò D. (2020). Glycogen Synthase Kinase-3 Signaling in Alzheimer's Disease. Biochim. Biophys. Acta Mol. Cel. Res. 1867 (5), 118664. 10.1016/j.bbamcr.2020.118664 PMC7047718 32006534

[B36] Lee S. J. Chung Y. H. Joo K. M. Lim H. C. Jeon G. S. Kim D. (2006). Age-related Changes in Glycogen Synthase Kinase 3beta (GSK3beta) Immunoreactivity in the Central Nervous System of Rats. Neurosci. Lett. 409 (2), 134–139. 10.1016/j.neulet.2006.09.026 17046157

[B37] Lei W. Deng Y. F. Hu X. Y. Ni J. N. Jiang M. Bai G. (2019). Phthalides, Senkyunolide A and Ligustilide, Show Immunomodulatory Effect in Improving Atherosclerosis, through Inhibiting AP-1 and NF-Κb Expression. Biomed. Pharmacother. 117, 109074. 10.1016/j.biopha.2019.109074 31177061

[B38] Li H. L. Wang H. H. Liu S. J. Deng Y. Q. Zhang Y. J. Tian Q. (2007). Phosphorylation of Tau Antagonizes Apoptosis by Stabilizing Beta-Catenin, a Mechanism Involved in Alzheimer's Neurodegeneration. Proc. Natl. Acad. Sci. U S A. 104 (9), 3591–3596. 10.1073/pnas.0609303104 17360687 PMC1805527

[B39] Li W. Tang Y. Chen Y. Duan J. A. (2012). Advances in the Chemical Analysis and Biological Activities of Chuanxiong. Molecules 17 (9), 10614–10651. 10.3390/molecules170910614 22955453 PMC6268834

[B40] Li W. Tang Y. Qian Y. Shang E. Wang L. Zhang L. (2014). Comparative Analysis of Main Aromatic Acids and Phthalides in Angelicae Sinensis Radix, Chuanxiong Rhizoma, and Fo-Shou-San by a Validated UHPLC-TQ-MS/MS. J. Pharm. Biomed. Anal. 99, 45–50. 10.1016/j.jpba.2014.07.007 25061713

[B41] Liao S. L. Kao T. K. Chen W. Y. Lin Y. S. Chen S. Y. Raung S. L. (2004). Tetramethylpyrazine Reduces Ischemic Brain Injury in Rats. Neurosci. Lett. 372 (1-2), 40–45. 10.1016/j.neulet.2004.09.013 15531085

[B42] Lin J. Hao C. Gong Y. Zhang Y. Li Y. Feng Z. (2021). Effect of Tetramethylpyrazine on Neuroplasticity after Transient Focal Cerebral Ischemia Reperfusion in Rats. Evid. Based Complement. Alternat Med. 2021, 1587241. 10.1155/2021/1587241 33531914 PMC7834793

[B43] Liu Y. Ding R. Xu Z. Xue Y. Zhang D. Zhang Y. (2021). Roles and Mechanisms of the Protein Quality Control System in Alzheimer's Disease. Int. J. Mol. Sci. 23 (1). 10.3390/ijms23010345 PMC8745298 35008771

[B44] Lu F. Li X. Li W. Wei K. Yao Y. Zhang Q. (2017). Tetramethylpyrazine Reverses Intracerebroventricular Streptozotocin-Induced Memory Deficits by Inhibiting GSK-3β. Acta Biochim. Biophys. Sin (Shanghai) 49 (8), 722–728. 10.1093/abbs/gmx059 28633346

[B45] Lucas J. J. Hernández F. Gómez-Ramos P. Morán M. A. Hen R. Avila J. (2001). Decreased Nuclear Beta-Catenin, Tau Hyperphosphorylation and Neurodegeneration in GSK-3beta Conditional Transgenic Mice. EMBO J. 20 (1-2), 27–39. 10.1093/emboj/20.1.27 11226152 PMC140191

[B46] Marongiu B. Piras A. Porcedda S. Falconieri D. Maxia A. Frau M. A. (2013). Isolation of the Volatile Fraction from Apium graveolens L. (Apiaceae) by Supercritical Carbon Dioxide Extraction and Hydrodistillation: Chemical Composition and Antifungal Activity. Nat. Prod. Res. 27 (17), 1521–1527. 10.1080/14786419.2012.725402 22974401

[B47] Mi H. Guo N. Kejariwal A. Thomas P. D. (2007). PANTHER Version 6: Protein Sequence and Function Evolution Data with Expanded Representation of Biological Pathways. Nucleic Acids Res. 35 (Database issue), D247–D252. 10.1093/nar/gkl869 17130144 PMC1716723

[B48] Momin R. A. Nair M. G. (2002). Antioxidant, Cyclooxygenase and Topoisomerase Inhibitory Compounds from Apium graveolens Linn. Seeds. Phytomedicine 9 (4), 312–318. 10.1078/0944-7113-00131 12120812

[B49] Moore A. M. Mahoney E. Dumitrescu L. De Jager P. L. Koran M. E. I. Petyuk V. A. (2020). APOE ε4-specific Associations of VEGF Gene Family Expression with Cognitive Aging and Alzheimer's Disease. Neurobiol. Aging 87, 18–25. 10.1016/j.neurobiolaging.2019.10.021 31791659 PMC7064375

[B50] Nam K. N. Kim K. P. Cho K. H. Jung W. S. Park J. M. Cho S. Y. (2013). Prevention of Inflammation-Mediated Neurotoxicity by Butylidenephthalide and its Role in Microglial Activation. Cell Biochem. Funct. 31 (8), 707–712. 10.1002/cbf.2959 23400915

[B51] Qu X. Guan P. Han L. Wang Z. Huang X. (2021). Levistolide A Attenuates Alzheimer's Pathology through Activation of the PPARγ Pathway. Neurotherapeutics 18 (1), 326–339. 10.1007/s13311-020-00943-1 33034847 PMC8116477

[B52] Quiroz Y. T. Sperling R. A. Norton D. J. Baena A. Arboleda-Velasquez J. F. Cosio D. (2018). Association between Amyloid and Tau Accumulation in Young Adults with Autosomal Dominant Alzheimer Disease. JAMA Neurol. 75 (5), 548–556. 10.1001/jamaneurol.2017.4907 29435558 PMC5885174

[B53] Rabinovici G. D. (2021). Controversy and Progress in Alzheimer's Disease - FDA Approval of Aducanumab. N. Engl. J. Med. 385 (9), 771–774. 10.1056/NEJMp2111320 34320284

[B54] Ru J. Li P. Wang J. Zhou W. Li B. Huang C. (2014). TCMSP: A Database of Systems Pharmacology for Drug Discovery from Herbal Medicines. J. Cheminform 6, 13. 10.1186/1758-2946-6-13 24735618 PMC4001360

[B55] Sakai S. Ochiai H. Nakajima K. Terasawa K. (1997). Inhibitory Effect of Ferulic Acid on Macrophage Inflammatory Protein-2 Production in a Murine Macrophage Cell Line, RAW264.7. Cytokine 9 (4), 242–248. 10.1006/cyto.1996.0160 9112332

[B56] Shannon P. Markiel A. Ozier O. Baliga N. S. Wang J. T. Ramage D. (2003). Cytoscape: a Software Environment for Integrated Models of Biomolecular Interaction Networks. Genome Res. 13 (11), 2498–2504. 10.1101/gr.1239303 14597658 PMC403769

[B57] Shi Y. Cai E. L. Yang C. Ye C. Y. Zeng P. Wang X. M. (2020). Protection of Melatonin against Acidosis-Induced Neuronal Injuries. J. Cel. Mol. Med. 24 (12), 6928–6942. 10.1111/jcmm.15351 PMC7299701 32364678

[B58] Sundelöf J. Sundström J. Hansson O. Eriksdotter-Jönhagen M. Giedraitis V. Larsson A. (2010). Higher Cathepsin B Levels in Plasma in Alzheimer's Disease Compared to Healthy Controls. J. Alzheimers Dis. 22 (4), 1223–1230. 10.3233/JAD-2010-101023 20930303

[B59] Szklarczyk D. Gable A. L. Lyon D. Junge A. Wyder S. Huerta-Cepas J. (2019). STRING V11: Protein-Protein Association Networks with Increased Coverage, Supporting Functional Discovery in Genome-wide Experimental Datasets. Nucleic Acids Res. 47 (D1), D607–D613. 10.1093/nar/gky1131 30476243 PMC6323986

[B60] Tsukamoto T. Ishikawa Y. Miyazawa M. (2005). Larvicidal and Adulticidal Activity of Alkylphthalide Derivatives from Rhizome of Cnidium Officinale against Drosophila melanogaster. J. Agric. Food Chem. 53 (14), 5549–5553. 10.1021/jf050110v 15998112

[B61] Wang C. Zhang F. Jiang S. Siedlak S. L. Shen L. Perry G. (2016). Estrogen Receptor-α Is Localized to Neurofibrillary Tangles in Alzheimer's Disease. Sci. Rep. 6, 20352. 10.1038/srep20352 26837465 PMC4738266

[B62] Wang X. Yao Y. An C. Li X. Xiang F. Dong Y. (2020). Simultaneous Determination of 20 Bioactive Components in Chuanxiong Rhizoma from Different Production Origins in Sichuan Province by Ultra-high-performance Liquid Chromatography Coupled with Triple Quadrupole Mass Spectrometry Combined with Multivariate Statistical Analysis. Electrophoresis 41 (18-19), 1606–1616. 10.1002/elps.202000082 32557720

[B63] Wang X. M. Zeng P. Fang Y. Y. Zhang T. Tian Q. (2021). Progranulin in Neurodegenerative Dementia. J. Neurochem. 158 (2), 119–137. 10.1111/jnc.15378 33930186

[B64] Wang Y. Martinez-Vicente M. Krüger U. Kaushik S. Wong E. Mandelkow E. M. (2009). Tau Fragmentation, Aggregation and Clearance: the Dual Role of Lysosomal Processing. Hum. Mol. Genet. 18 (21), 4153–4170. 10.1093/hmg/ddp367 19654187 PMC2758146

[B65] Wang Y. Zhang Y. Wang Y. Shu X. Lu C. Shao S. (2021). Using Network Pharmacology and Molecular Docking to Explore the Mechanism of Shan Ci Gu (Cremastra Appendiculata) against Non-small Cell Lung Cancer. Front. Chem. 9, 682862. 10.3389/fchem.2021.682862 34178945 PMC8220148

[B66] Wang Z. Sun H. Yao X. Li D. Xu L. Li Y. (2016). Comprehensive Evaluation of Ten Docking Programs on a Diverse Set of Protein-Ligand Complexes: the Prediction Accuracy of Sampling Power and Scoring Power. Phys. Chem. Chem. Phys. 18 (18), 12964–12975. 10.1039/c6cp01555g 27108770

[B67] Wen Y. Q. Fu Y. Wei J. P. Chen H. Dai Y. Xu S. J. (2018). Study on the Difference of Prescriptions for the Treatment of Alzheimer's Disease and Vascular Dementia Based on the Chinese Medicine Heritage Auxiliary Platform. World Sci. Technology/Modernization Traditional Chin. Med. Materia Med. 20 (12), 2150–2155.

[B68] Westbrook J. Feng Z. Jain S. Bhat T. N. Thanki N. Ravichandran V. (2002). The Protein Data Bank: Unifying the Archive. Nucleic Acids Res. 30 (1), 245–248. 10.1093/nar/30.1.245 11752306 PMC99110

[B69] Xia Y. Prokop S. Giasson B. I. (2021). "Don't Phos over Tau": Recent Developments in Clinical Biomarkers and Therapies Targeting Tau Phosphorylation in Alzheimer's Disease and Other Tauopathies. Mol. Neurodegener. 16 (1), 37. 10.1186/s13024-021-00460-5 34090488 PMC8180161

[B70] Xie Q. Zhang L. Xie L. Zheng Y. Liu K. Tang H. (2020). Z-ligustilide: A Review of its Pharmacokinetics and Pharmacology. Phytother. Res. 34 (8), 1966–1991. 10.1002/ptr.6662 32135035

[B71] Xiong G. Wu Z. Yi J. Fu L. Yang Z. Hsieh C. (2021). ADMETlab 2.0: an Integrated Online Platform for Accurate and Comprehensive Predictions of ADMET Properties. Nucleic Acids Res. 49 (W1), W5–W14. 10.1093/nar/gkab255 33893803 PMC8262709

[B72] Xu M. Zhang D. F. Luo R. Wu Y. Zhou H. Kong L. L. (2018). A Systematic Integrated Analysis of Brain Expression Profiles Reveals YAP1 and Other Prioritized Hub Genes as Important Upstream Regulators in Alzheimer's Disease. Alzheimers Dement 14 (2), 215–229. 10.1016/j.jalz.2017.08.012 28923553

[B73] Xu W. Yang L. Li J. (2016). Protection against β-amyloid-induced Neurotoxicity by Naturally Occurring Z-Ligustilide through the Concurrent Regulation of p38 and PI3-K/Akt Pathways. Neurochem. Int. 100, 44–51. 10.1016/j.neuint.2016.08.012 27580711

[B74] Yang W. T. Zheng X. W. Chen S. Shan C. S. Xu Q. Q. Zhu J. Z. (2017). Chinese Herbal Medicine for Alzheimer's Disease: Clinical Evidence and Possible Mechanism of Neurogenesis. Biochem. Pharmacol. 141, 143–155. 10.1016/j.bcp.2017.07.002 28690138

[B75] Yin D. D. Wang Y. L. Yang M. Yin D. K. Wang G. K. Xu F. (2019). Analysis of Chuanxiong Rhizoma Substrate on Production of Ligustrazine in Endophytic Bacillus Subtilis by Ultra High Performance Liquid Chromatography with Quadrupole Time-Of-Flight Mass Spectrometry. J. Sep. Sci. 42 (19), 3067–3076. 10.1002/jssc.201900030 31347249

[B76] Yin D. D. Yang M. Wang Y. L. Yin D. K. Liu H. K. Zhou M. (2018). High Tetramethylpyrazine Production by the Endophytic Bacterial Bacillus Subtilis Isolated from the Traditional Medicinal Plant Ligusticum Chuanxiong Hort. AMB Express 8 (1), 193. 10.1186/s13568-018-0721-1 30564983 PMC6298913

[B77] Yu G. Wang L. G. Han Y. He Q. Y. (2012). clusterProfiler: an R Package for Comparing Biological Themes Among Gene Clusters. OMICS 16 (5), 284–287. 10.1089/omi.2011.0118 22455463 PMC3339379

[B78] Yu G. Wang L. G. Yan G. R. He Q. Y. (2015). DOSE: an R/Bioconductor Package for Disease Ontology Semantic and Enrichment Analysis. Bioinformatics 31 (4), 608–609. 10.1093/bioinformatics/btu684 25677125

[B79] Zeng P. Wang X. M. Ye C. Y. Su H. F. Fang Y. Y. Zhang T. (2021a). Mechanistic Insights into the Anti-depressant Effect of Emodin: an Integrated Systems Pharmacology Study and Experimental Validation. Aging (Albany NY) 13 (11), 15078–15099. 10.18632/aging.203072 34051074 PMC8221295

[B80] Zeng P. Wang X. M. Ye C. Y. Su H. F. Tian Q. (2021b). The Main Alkaloids in Uncaria Rhynchophylla and Their Anti-alzheimer's Disease Mechanism Determined by a Network Pharmacology Approach. Int. J. Mol. Sci. 22 (7). 10.3390/ijms22073612 PMC8036964 33807157

[B81] Zeng P. Yi Y. Su H. F. Ye C. Y. Sun Y. W. Zhou X. W. (2021c). Key Phytochemicals and Biological Functions of Chuanxiong Rhizoma against Ischemic Stroke: A Network Pharmacology and Experimental Assessment. Front. Pharmacol. 12, 758049. 10.3389/fphar.2021.758049 34992531 PMC8724589

[B82] Zhang H. Cao Y. Ma L. Wei Y. Li H. (2021). Possible Mechanisms of Tau Spread and Toxicity in Alzheimer's Disease. Front. Cel. Dev. Biol. 9, 707268. 10.3389/fcell.2021.707268 PMC8355602 34395435

[B83] Zhang Z. Wei T. Hou J. Li G. Yu S. Xin W. (2003). Iron-induced Oxidative Damage and Apoptosis in Cerebellar Granule Cells: Attenuation by Tetramethylpyrazine and Ferulic Acid. Eur. J. Pharmacol. 467 (1-3), 41–47. 10.1016/s0014-2999(03)01597-8 12706453

[B84] Zheng Q. Wei S. F. Xiong W. H. Xue X. Yu J. Wu Z. F. (2013). Analysis on Application of Chuanxiong Rhizomain Chinese Patent Medicine Formula for Treating Headache. Chin. Traditional Herbal Drugs 44 (19), 2777–2781.

[B85] Zhou X. F. Zhou W. E. Liu W. J. Luo M. J. Wu X. Q. Wang Y. (2021). A Network Pharmacology Approach to Explore the Mechanism of HuangZhi YiShen Capsule for Treatment of Diabetic Kidney Disease. J. Transl. Int. Med. 9 (2), 98–113. 10.2478/jtim-2021-0020 34497749 PMC8386324

[B86] Zhu T. Wang L. Feng Y. Sun G. Sun X. (2021). Classical Active Ingredients and Extracts of Chinese Herbal Medicines: Pharmacokinetics, Pharmacodynamics, and Molecular Mechanisms for Ischemic Stroke. Oxid. Med. Cel. Longev. 2021 (7), 8868941. 10.1155/2021/8868941 PMC7984881 33791075

[B87] Zong X. Ji X. M. Wei F. Q. Shi Z. R. (2014). Analysis on Prescription Rules of Treating Senile Dementia Based on Traditional Chinese Medicine Inheritance Auxiliary Systems. Zhongguo Zhong Yao Za Zhi 39 (4), 640–643. 25204138

[B88] Zuo A. Wang L. Xiao H. Li L. Liu Y. Yi J. (2011). Identification of the Absorbed Components and Metabolites in Rat Plasma after Oral Administration of Rhizoma Chuanxiong Decoction by HPLC-ESI-MS/MS. J. Pharm. Biomed. Anal. 56 (5), 1046–1056. 10.1016/j.jpba.2011.08.010 21880453

