# Supplementary figures and images for: A Tau Pathogenesis-Based Network Pharmacology Approach for Exploring the Protections of Chuanxiong Rhizoma in Alzheimer’s Disease
Source: Front Pharmacol. 2022 Apr 21;13:877806. doi: 10.3389/fphar.2022.877806 (PMC9068950; doi:10.3389/fphar.2022.877806)

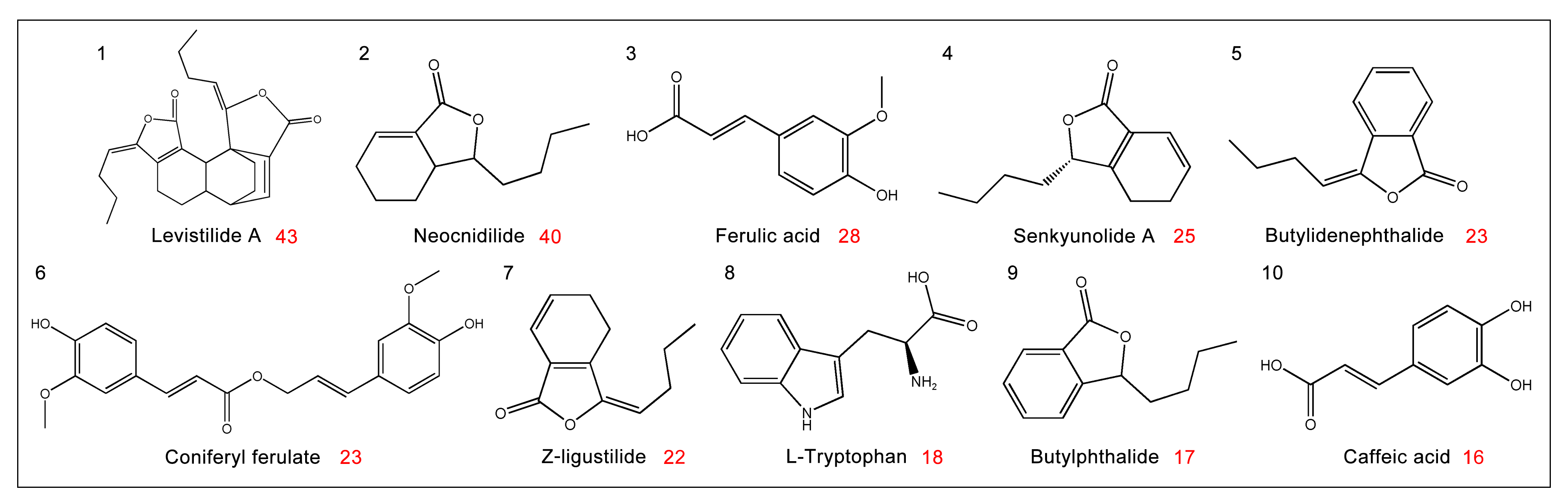

Supplement: Supplementary file 1 [file Image3.TIF]

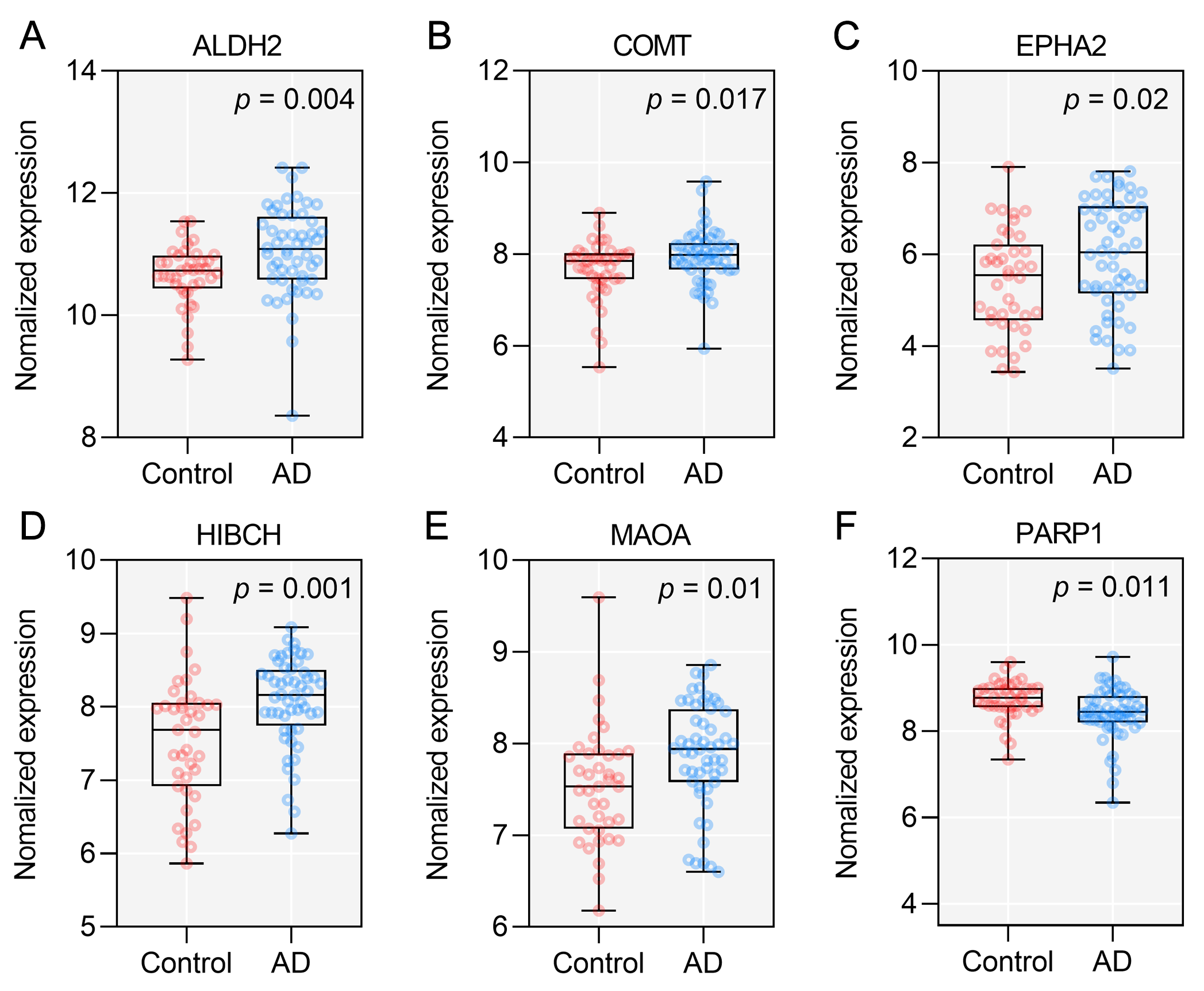

Supplement: Supplementary file 2 [file Image4.TIF]

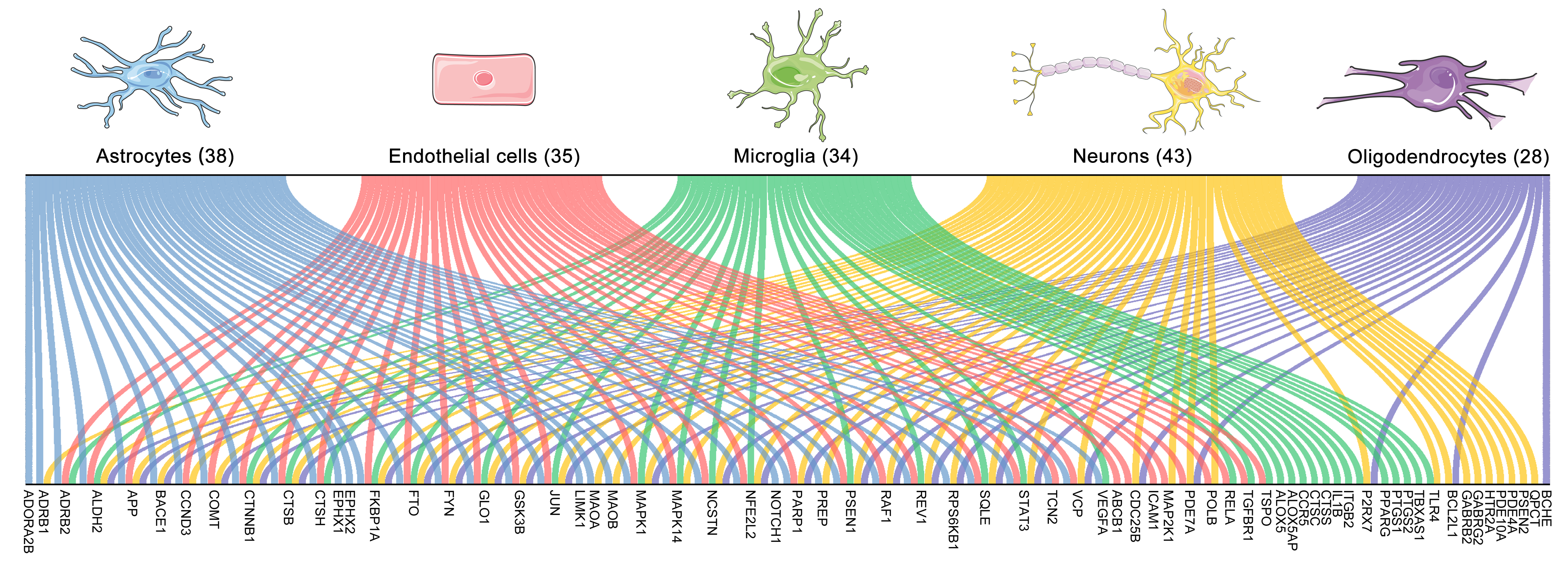

Supplement: Supplementary file 3 [file Image2.TIF]

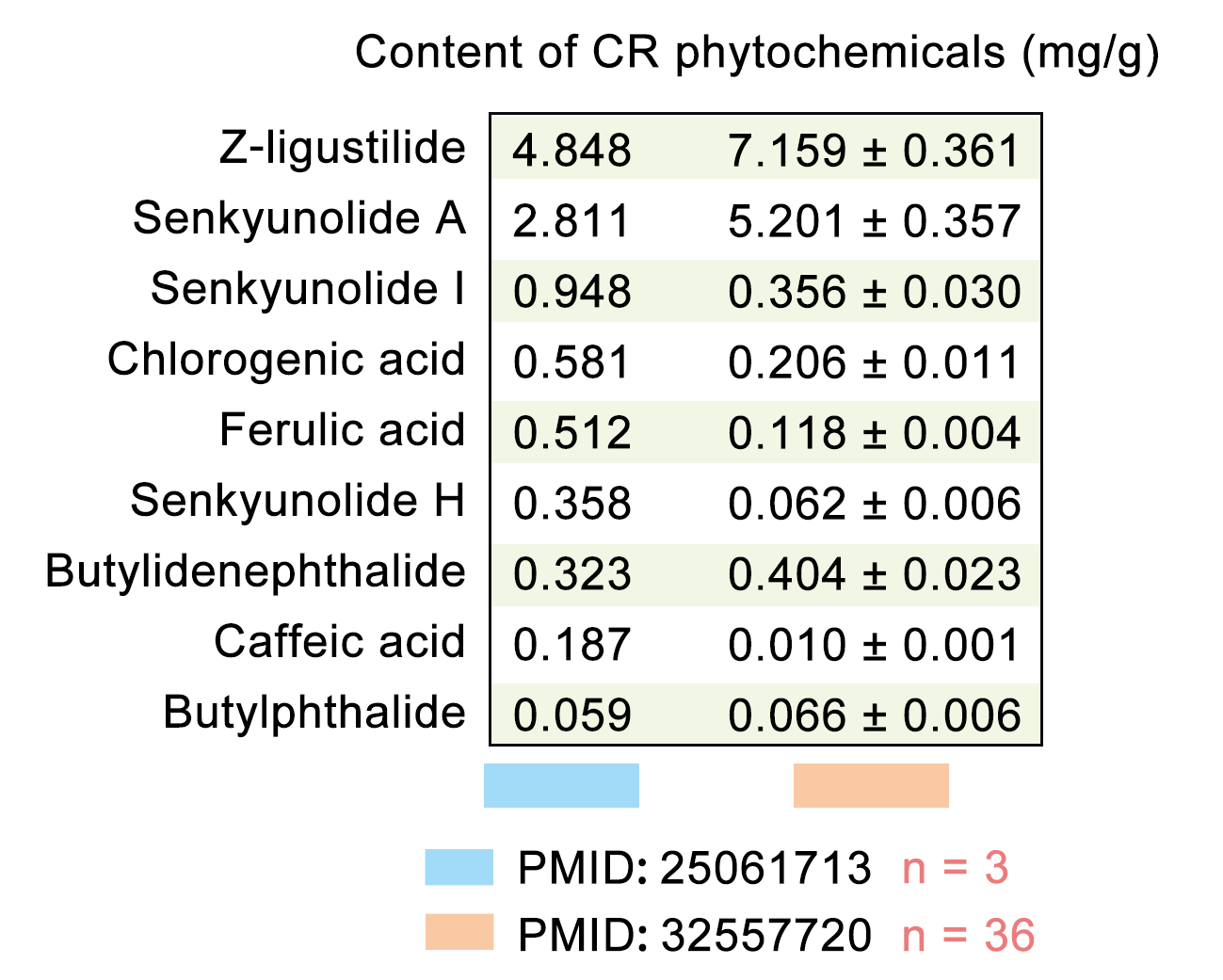

Supplement: Supplementary file 4 [file Image1.TIF]
